# Niche-specific adaptation of *Lactobacillus helveticus* strains isolated from malt whisky and dairy fermentations

**DOI:** 10.1099/mgen.0.000560

**Published:** 2021-04-26

**Authors:** Yoshihiko Kido, Shintaro Maeno, Hiroki Tanno, Yuko Kichise, Yuh Shiwa, Akihito Endo

**Affiliations:** ^1^​ Department of Food, Aroma and Cosmetic Chemistry, Tokyo University of Agriculture, Hokkaido 099-2493, Japan; ^2^​ NODAI Genome Research Center, Tokyo University of Agriculture, Tokyo 156-8502, Japan; ^3^​ Department of Molecular Microbiology, Faculty of Life Sciences, Tokyo University of Agriculture, Tokyo 156-8502, Japan

**Keywords:** carbohydrate metabolism, dairy fermentation, glycoside hydrolases, *L. helveticus*, stress tolerance, whisky fermentation

## Abstract

*
Lactobacillus helveticus* is a well characterized lactobacillus for dairy fermentations that is also found in malt whisky fermentations. The two environments contain considerable differences related to microbial growth, including the presence of different growth inhibitors and nutrients. The present study characterized *L. helveticus* strains originating from dairy fermentations (called milk strains hereafter) and malt whisky fermentations (called whisky strains hereafter) by *in vitro* phenotypic tests and comparative genomics. The whisky strains can tolerate ethanol more than the milk strains, whereas the milk strains can tolerate lysozyme and lactoferrin more than the whisky strains. Several plant-origin carbohydrates, including cellobiose, maltose, sucrose, fructooligosaccharide and salicin, were generally metabolized only by the whisky strains, whereas milk-derived carbohydrates, i.e. lactose and galactose, were metabolized only by the milk strains. Milk fermentation properties also distinguished the two groups. The general genomic characteristics, including genomic size, number of coding sequences and average nucleotide identity values, differentiated the two groups. The observed differences in carbohydrate metabolic properties between the two groups correlated with the presence of intact specific enzymes in glycoside hydrolase (GH) families GH1, GH4, GH13, GH32 and GH65. Several GHs in the milk strains were inactive due to the presence of stop codon(s) in genes encoding the GHs, and the inactivation patterns of the genes encoding specific enzymes assigned to GH1 in the milk strains suggested a possible diversification manner of *L. helveticus* strains. The present study has demonstrated how *L. helveticus* strains have adapted to their habitats.

## Data Summary

The genomic data of the *Lactobacillus helveticus* strains determined in the present study were deposited into GenBank/EMBL/DDBJ under accession numbers BLYW01000000 (JCM 1120^T^), BLYS01000000 (JCM 1005), BLYT01000000 (JCM 1006), BLYU01000000 (JCM 1007), BLYV01000000 (JCM 1062), BLYX01000000 (JCM 20397), BLYO01000000 (H-8), BLYR01000000 (LMG 22465), BLYP01000000 (W-6) and BLYQ01000000 (Y-10).

**Impact Statement**

*
Lactobacillus helveticus* is one of the most important micro-organisms in the dairy industry and is also important in malt whisky fermentations. The present study demonstrated that *L. helveticus* strains are phenotypically and genomically well adapted to their habitats, and the whisky strains exhibit unique characteristics when compared with previous knowledge of *L. helveticus *. Carbohydrate metabolic properties clearly distinguished the *L. helveticus* strains based on their origins. The whisky strains are richer glycoside hydrolase (GH) holders than the milk strains, and GH profiling was consistent with their carbohydrate metabolic properties. The milk strains contained several genes encoding GHs in inactive forms due to the presence of stop codons or partial deletions, which may be due to insertion-sequence-associated gene decay. Inactivation patterns of genes encoding GH1 enzymes suggested a possible diversification manner of *L. helveticus* strains. The present study also found interesting milk strains possessing both milk-strain-specific and whisky-strain-specific genomic characteristics.

## Introduction

*Lactobacillus helveticus* was originally isolated from an emmental cheese and can be found in several natural dairy fermentations, including cheeses [1], kefir grains [2], Indian fermented milks [3], fermented mare’s milk (airag) [4], fermented camel/cow/goat/yak milk (khormog/tarag) [5], Ghanan fermented unpasteurized milk (nunu) [6] and natural whey cultures [7]. The organism is thermophilic and possesses strong protease activity and, thus, is used as a starter for several dairy fermentations [8]. Proteolysis results in the accumulation of favourable flavour and functional peptides in the products [9]. Potential probiotic properties of the organism were previously demonstrated in animal and clinical studies [10–12]. Therefore, *L. helveticus* is one of the most industrially important organisms in the lactic acid bacteria group, especially in the dairy industry.

*Lactobacillus suntoryeus* was originally isolated from fermentation samples of malt whisky distilleries in Japan and Scotland [13]. The organism, which was described as *Lactobacillus* sp. in the previous article, was less abundant at the beginning of the malt whisky fermentation, but dominated after 70 h [14]. Strain H-8 in the species, although it was described as *Lactobacillus crispatus* in the article [15], was capable of decarboxylation of *p*-coumaric acid to 4-vinylphenol [15], which is potentially beneficial for the quality of malt whisky, suggesting that *L. suntoryeus* is well adapted to whisky fermentation. However, *L. suntoryeus* was later reclassified as a heterotypic synonym of *L. helveticus* [16], suggesting that *L. helveticus* is not only a specific organism to dairy fermentations, but also an important microbe for malt whisky fermentation.

Malt juices and milks have marked differences relevant for the growth of *L. helveticus *. For example, the major carbohydrates available are maltose and maltotriose in malt whisky fermentations [14], and lactose in milks. Free amino acids are available in the whisky fermentations [17], whereas milks contain trace free amino acids but are rich in casein proteins [18]. The ethanol concentration exceeds 8 % (v/v) during whisky fermentation [14], but the accumulation of ethanol is noted for only a few milk fermentations, e.g. airag (normally <3 %) [4]. Milks contain microbial growth inhibition substances, including lysozyme and lactoferrin [19]. Thus, *L. helveticus* strains may have adapted to the physiologically different habitats and obtained specific characteristics to survive in their habitats. Recent studies revealed that lactic acid bacteria adapted to each habitat at the genomic level, and obtained or lost genes during the adaptation [20–22].

In the present study, five strains of *L. helveticus* originating from malt whisky fermentations (called whisky strains hereafter) and six strains of *L. helveticus* originating from milk fermentations (called milk strains hereafter) were included in physiological tests to clarify their niche-specific phenotypic characteristics. Draft genome sequences of the strains from the two origins were determined and used to study their adaptation at the genomic level. Moreover, complete genome sequences of eight strains of *L. helveticus* originating from milk fermentations were obtained from a public database and included in the genomic analysis. The present study revealed that *L. helveticus* has obtained niche-specific phenotypic characteristics and has undergone niche-specific evolution at the genomic level.

## Methods

### Bacterial strains and culture conditions

Five whisky strains (JCM 30915, LMG 22465, H-8, W-6 and Y-10) and six milk strains (JCM 1120^T^, JCM 1005, JCM 1006, JCM 1007, JCM 1062 and JCM 20397) classified as *L. helveticus* were included for *in vitro* phenotypic characterization of strains originating from different habitats (Table 1). Strains H-8, W-6 and Y-10 were obtained from Suntory, and the other strains were obtained from public culture collections. Of the five whisky strains, LMG 22465 originated from a distillery in Scotland, and the remaining four were from different distilleries in Japan [13]. *L. helveticus* strains were cultured in MRS broth supplemented with 0.05 % (w/v) l-cysteine-HCl (mMRS broth) at 37 °C for 24 h.

**Table 1.** *
L. helveticus* strains used in the present study

Strain type and characterization	Strain	Source	ID/accession no.	Genome status	No. of sequences	Genome size (Mbp)	No. of CDSs	Completeness (%)*		Note
								**Genus level**	**Species level**	
**Milk strains**										
Included in phenotypic and genomic characterization	JCM 1120^T^	Emmental cheese	BLYW01000000	Draft	412	2.12	2081	98.2	99.0	Type strain
	JCM 1005	Yoghurt	BLYS01000000	Draft	507	2.20	2146	98.9	96.2	Former *Lactobacillus bulgaricus *
	JCM 1006	Yoghurt	BLYT01000000	Draft	318	2.02	2037	98.9	98.2	Former *Lactobacillus bulgaricus *
	JCM 1007	Swiss cheese	BLYU01000000	Draft	495	2.28	2237	98.9	97.6	
	JCM 1062	Dairy product	BLYV01000000	Draft	459	2.27	2243	98.9	97.6	
	JCM 20397	Raw milk	BLYX01000000	Draft	302	2.02	2042	98.9	98.2	Former *Lactobacillus bulgaricus *
Included in genomic characterization	CAUH18	Koumiss	GCA_001308285.1	Complete	1	2.16	2235	98.7	98.2	
	CNRZ32	Cheese	GCA_000422165.1	Complete	1	2.23	2407	98.9	97.6	
	DPC 4571	Cheese	GCA_000015385.1	Complete	1	2.08	2199	98.6	99.0	
	H9	Kurut	GCA_000525715.1	Complete	1	1.87	2015	98.9	97.0	
	H10	Fermented milk	GCA_000189515.1	Complete	2	2.17	2265	98.9	99.0	
	KLDS1.8701	Sour milk	GCA_000961015.1	Complete	2	2.11	2255	98.9	97.6	
	MB2-1	Fermented milk	GCA_001006025.1	Complete	1	2.08	2337	98.9	98.3	
	R0052	Sweet acidophilus milk	GCA_000165775.3	Complete	1	2.13	2288	98.9	99.0	
**Whisky strains**										
Included in phenotypic and genomic characterization	H-8	Malt whisky fermentation	BLYO01000000	Draft	375	1.96	1922	98.2	90.8	Former *Lactobacillus suntoryeus *
	JCM 30912 (=LMG 22464)	Malt whisky fermentation	ERR387534	Draft	232	1.75	1825	97.6	89.2	Former *Lactobacillus suntoryeus* type strain
	LMG 22465	Malt whisky fermentation	BLYR01000000	Draft	655	1.92	1721	97.4	89.5	Former *Lactobacillus suntoryeus *
	W-6	Malt whisky fermentation	BLYP01000000	Draft	360	1.96	1904	98.2	90.8	Former *Lactobacillus suntoryeus *
	Y-10	Malt whisky fermentation	BLYQ01000000	Draft	369	1.97	1916	98.2	90.8	Former *Lactobacillus suntoryeus *

*Completeness was determined by the CheckM (v1.0.11) program. Completeness at the genus level was the ratio of stored genes in ubiquitous and single-copy genes within the genus *Lactobacillus*, and that at the species level was the gene ratio within *L. helveticus*, as set in the program.

### Carbohydrate metabolism, milk fermentation and enzyme activities

Carbohydrate fermentation reactions were initially tested using API CHL galleries (bioMérieux), according to the manufacturer’s instructions, but the whisky strains exhibited weak and slow growth in this test. Therefore, the carbohydrate metabolic properties were investigated using lactobacilli-carbohydrate test medium as described previously [23], with slight modification. The tested medium was composed of (w/v) 0.5 % yeast extract, 0.5 % polypeptone, 0.5 % tryptone, 0.5 % Lab-Lemco powder, 0.5 % sodium acetate, 0.2 % triammonium citrate, 0.05 % l-cysteine-HCl, 0.05 % Tween 80, 0.2 % K_2_HPO_4_, 0.02 % MgSO_4_.7H_2_O, 0.001 % MnSO_4_.4H_2_O, 0.001 % FeSO_4_.7H_2_O, 0.001 % NaCl and 1 % carbohydrate (pH 6.8). In this study, 21 carbohydrates, which are well metabolized in lactobacilli, were included, as shown in Table 2. The cultures were incubated at 37 °C for 48 h and growth was monitored every 24 h by measuring optical density at 660 nm with a spectrophotometer (model U-2800A; Hitachi). All strains had an OD_660_ >1.5 in the tested broth with glucose supplementation after 48 h of incubation. A positive reaction was defined as an OD_660_ >1 or relative biomass accumulation greater than 50 % compared with biomass accumulation on glucose after 48 h of incubation, whereas a weak reaction was defined as 0.5 <OD_660_ <1 or relative biomass accumulation between 30 and 50 % compared with biomass accumulation on glucose after 48 h of incubation.

**Table 2.** Carbohydrate metabolic properties of *L. helveticus* strains originating from dairy products and malt whisky fermentations Positive reactions were defined as an OD_660_ >1 or relative biomass accumulation greater than 50% compared with biomass accumulation on glucose after 48 h incubation, whereas weak reactions were defined as 0.5< OD_660_ < 1 or relative biomass accumulation between 30 and 50% compared with biomass accumulation on glucose after 48 h incubation. All strains showed negative reactions with l-arabinose, d-ribose, d-xylose, raffinose, sorbitol, melibiose and mannitol.

Strains	d-Glucose	d-Fructose	d-Mannose	d-Galactose	Lactose	Trehalose	Cellobiose	Salicin	Sucrose	1-Kestose	FOS	Maltose	Maltotriose	Starch
**Milk strains**														
JCM 1120^T^	+	w	w	+	+	+	–	–	–	–	–	+	+	+
JCM 1005	+	+	+	+	+	–	–	–	–	–	–	–	–	–
JCM 1006	+	w	+	+	+	–	–	–	–	–	–	–	–	–
JCM 1007	+	+	+	+	+	–	–	–	–	–	–	–	+	w
JCM 1062	+	+	+	+	+	–	–	–	–	–	–	–	+	w
JCM 20397	+	w	+	+	+	–	–	–	–	–	–	–	–	–
**Whisky strains**														
H-8	+	+	+	–	–	–	+	+	+	+	+	+	+	+
JCM 30912	+	+	+	–	–	–	+	+	+	+	+	+	+	+
LMG 22465	+	+	+	–	–	–	+	+	+	+	+	+	+	+
W-6	+	+	+	–	–	–	+	+	+	+	+	+	+	+
Y-10	+	w	+	–	–	–	+	+	+	+	+	+	+	+

w, weak reaction.

Milk fermentation properties were assessed in ultra-high temperature (UHT) sterilized milk and 10 % (w/v) skim milk. Pre-cultured cells were washed three times with 0.85 % (w/v) NaCl (saline), diluted 10 times with saline and inoculated at a volume of 1 % in the milk. The inoculated milks were incubated at 37 °C for 48 h. Visible coagulation was evaluated by inclination of test tubes and pH was measured using a pH meter every 24 h. Moreover, to confirm milk fermentation properties in the whisky strains, 10 % skim milk supplemented with (i) 1 % (w/v) glucose, (ii) 1 % glucose and 0.5 % (w/v) yeast extract (Beckton Dickinson), or (iii) 1 % glucose and 0.5 % (w/v) casitone (Beckton Dickinson) was used (Table 3). These experiments were performed in triplicate.

**Table 3.** Milk fermentation properties of *L. helveticus* strains pH values are shown (coagulation of milks) after 48 h incubation: +, coagulated; −, not coagulated.

Strain	UHT milk	Skim milk			
			+Glucose	+Glucose	+Glucose
				+Yeast extract	+Casitone
**Milk strains**					
JCM 1120^T^	5.1 (+)	3.5 (+)	nd	nd	nd
JCM 1005	4.4 (+)	4.2 (+)	nd	nd	nd
JCM 1006	3.5 (+)	3.4 (+)	3.6 (+)	3.5 (+)	3.7 (+)
JCM 1007	3.4 (+)	3.5 (+)	nd	nd	nd
JCM 1062	3.3 (+)	3.3 (+)	nd	nd	nd
JCM 20397	3.5 (+)	3.3 (+)	nd	nd	nd
**Whisky strains**					
H-8	6.3 (−)	6.4 (−)	6.3 (−)	4.6 (+)	6.1 (−)
JCM 30912	6.4 (−)	6.4 (−)	6.3 (−)	3.8 (+)	6.0 (−)
LMG 22465	6.6 (−)	6.3 (−)	6.3 (−)	4.5 (+)	6.0 (−)
W-6	6.5 (−)	6.4 (−)	6.3 (−)	4.3 (+)	6.2 (−)
Y-10	6.5 (−)	6.3 (−)	6.4 (−)	4.7 (+)	6.1 (−)

nd, Not determined.

### Stress tolerance

**Fig. 1.**
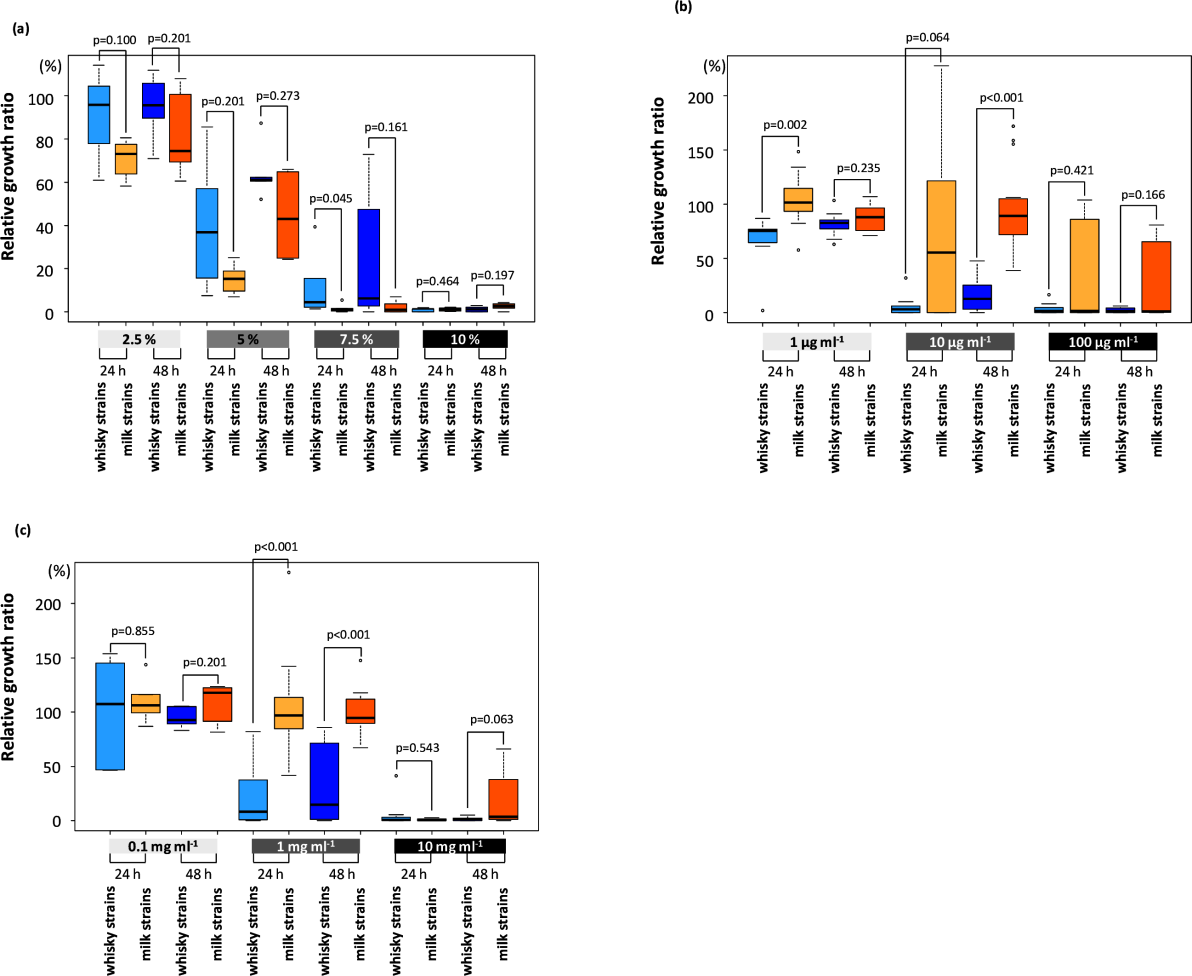
Ethanol tolerance (2.5, 5, 7.5 or 10 %) (**a**), lysozyme tolerance (1, 10 or 100 μg ml^−1^) (**b**), and lactoferrin tolerance (0.1, 1 or 10 mg ml^−1^) (**c**) of the whisky strains (*n*=5) and the milk strains (*n*=6). Relative growth ratios in mMRS broth supplemented with chemicals against the growth in mMRS broth without supplements after 24 and 48 h of incubation were measured. The line within each box represents the median, with the lower line as the 25 % border and the upper line as the 75 % border. The end of the upper vertical line represents the maximum data value; outliers were not considered. The end of the lower vertical line represents the lowest value; outliers were not considered. The separate dots indicate outliers. These experiments were performed in triplicate. The Mann–Whitney *U* test was applied to compare stress tolerances between the milk strains and whisky strains.

Stress tolerance of *L. helveticus* strains was characterized in mMRS broth supplemented with different concentrations of ethanol (2.5, 5, 7.5 % or 10 %; v/v), egg white lysozyme (Wako Chemicals; 1, 10 or 100 μg ml^−1^) and lactoferrin (Wako Chemicals; 100 μg ml^−1^, 1 mg ml^−1^ or 10 mg ml^−1^). mMRS broth without supplementation was included as a control. Pre-cultured cells were washed twice with saline, diluted 10 times with saline and inoculated at a volume of 1 % in the tested broth. The cultures were incubated at 37 °C for 48 h and growth was monitored every 24 h by measuring optical density at 660 nm with a spectrophotometer. Stress tolerance of the strains was evaluated by measurement of the relative cell biomass in the tested broth against that in the control broth (Fig. 1). These experiments were performed in triplicate.

### Draft genome sequencing and acquisition of genomic data of the reference strains

**Fig. 2.**
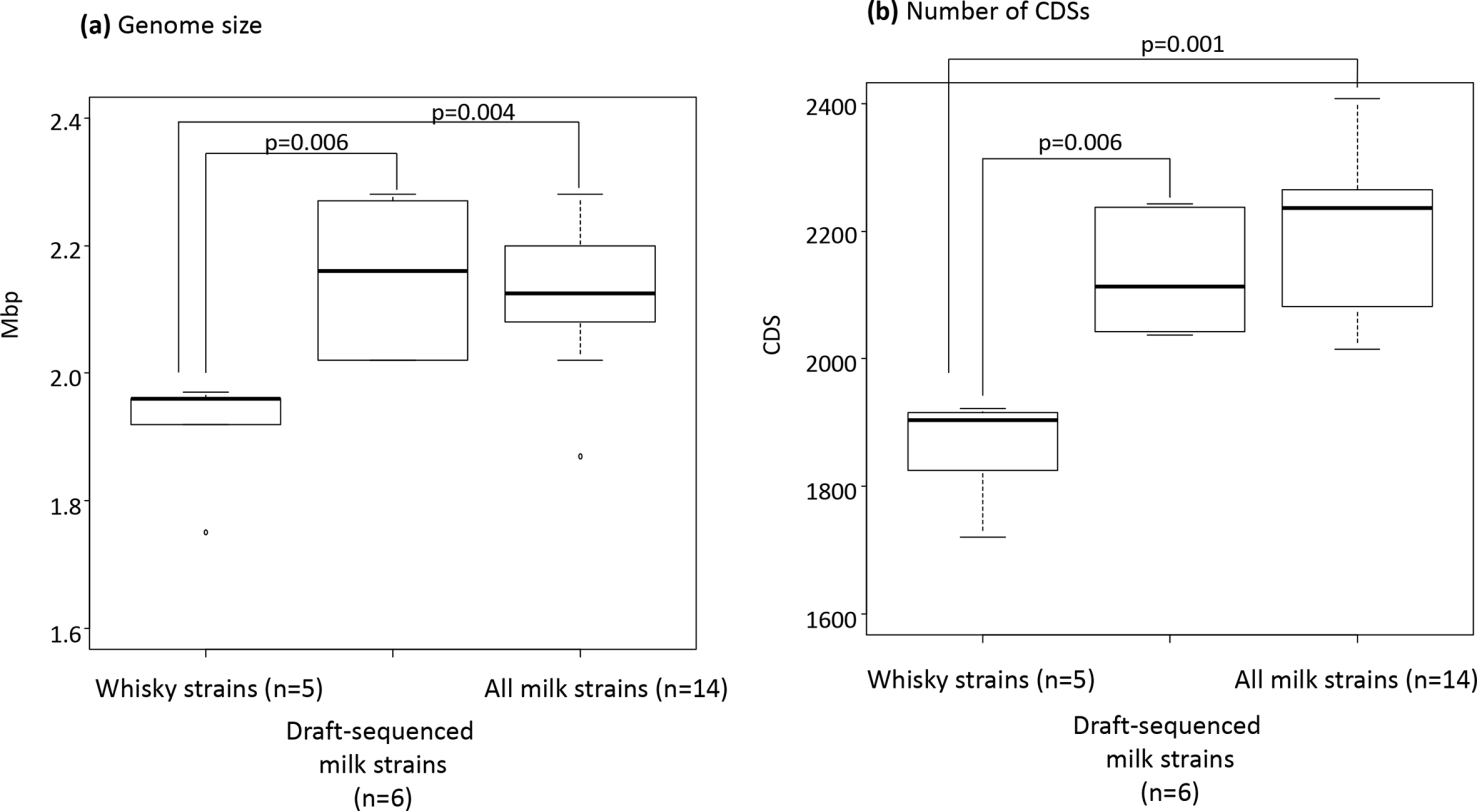
Genome sizes and numbers of CDSs in the whisky strains (*n*=5), all milk strains (*n*=14) and draft-sequenced milk strains (*n*=6). The line within each box represents the median, with the lower line as the 25 % border and the upper line as the 75 % border. The end of the upper vertical line represents the maximum data value; outliers were not considered. The end of the lower vertical line represents the lowest value; outliers were not considered. The separate dots indicate outliers. The Mann–Whitney *U* test was applied to compare between the whisky strains and all milk strains, and between the whisky strains and the draft-sequenced milk strains.

Whole-genome sequencing was conducted on the Illumina MiSeq, except that Illumina NovaSeq 6000 was used for sequencing of LMG 22465. Reads were assembled using Platanus_B (version 1.1.0) [24] with default settings. Sequences shorter than 300 bp were eliminated. The genome was annotated using the DDBJ Fast Annotation and Submission Tool (dfast, https://dfast.nig.ac.jp) [25]. The strains included in the *in vitro* phenotypic characterization were included in whole-genome sequencing, except that genomic data of JCM 30912 were obtained from the *Lactobacillus *-specific genome repository dfast Archive of Genome Annotation (DAGA, https://dfast.nig.ac.jp) [25]. The complete genome sequences of the eight strains of *L. helveticus *, which were all complete genomic data of *L. helveticus* strains available in the DAGA at the time of analysis (December 2018), were also obtained and included in the comparative genomic analysis (Table 1, Fig. 2).

### Genome analysis

**Fig. 3.**
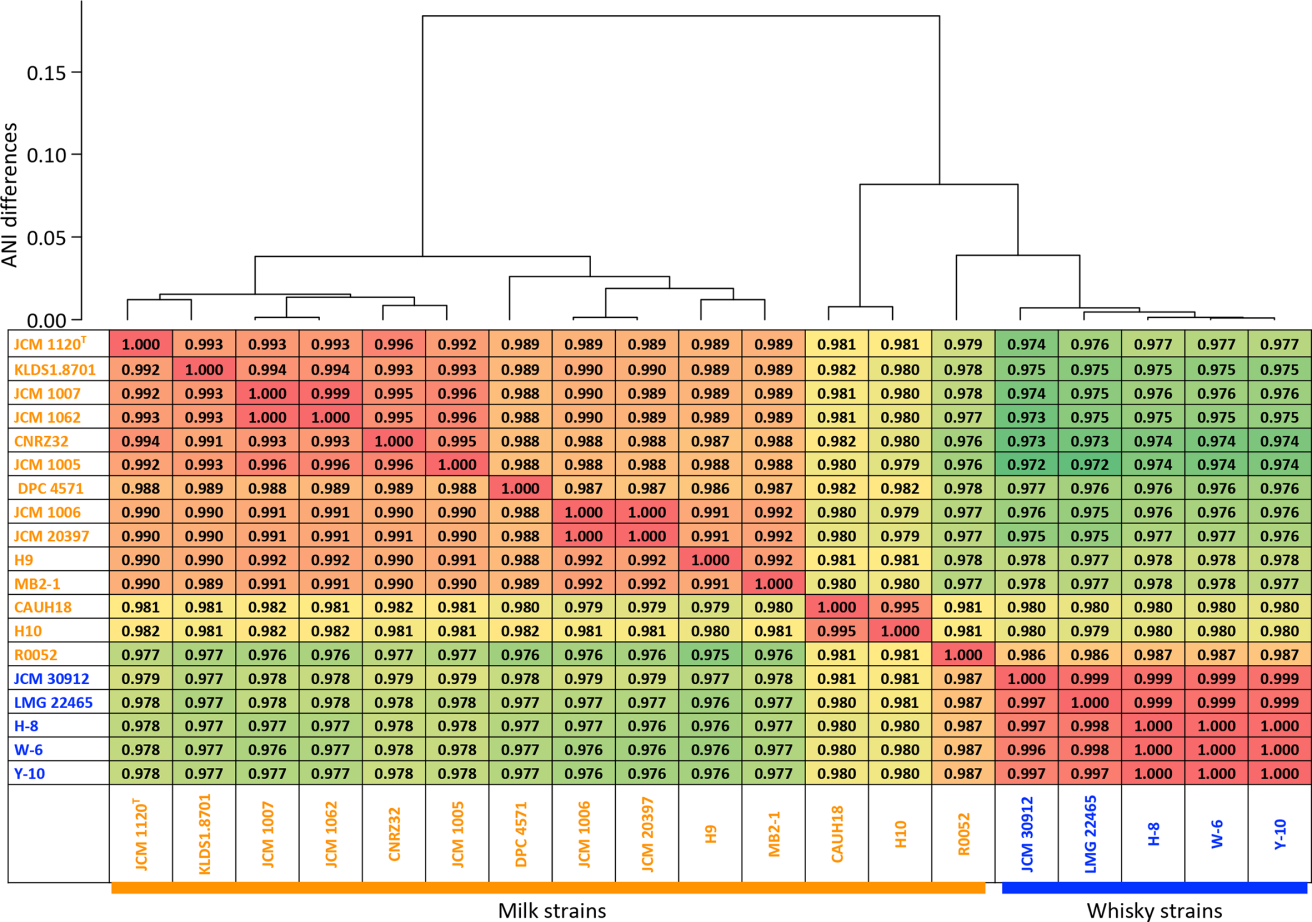
ANI values and ANI-value-based hierarchical clustering of *L. helveticus* strains. The strain names of the whisky strains are indicated in blue and those of the milk strains are in orange.

**Fig. 4.**
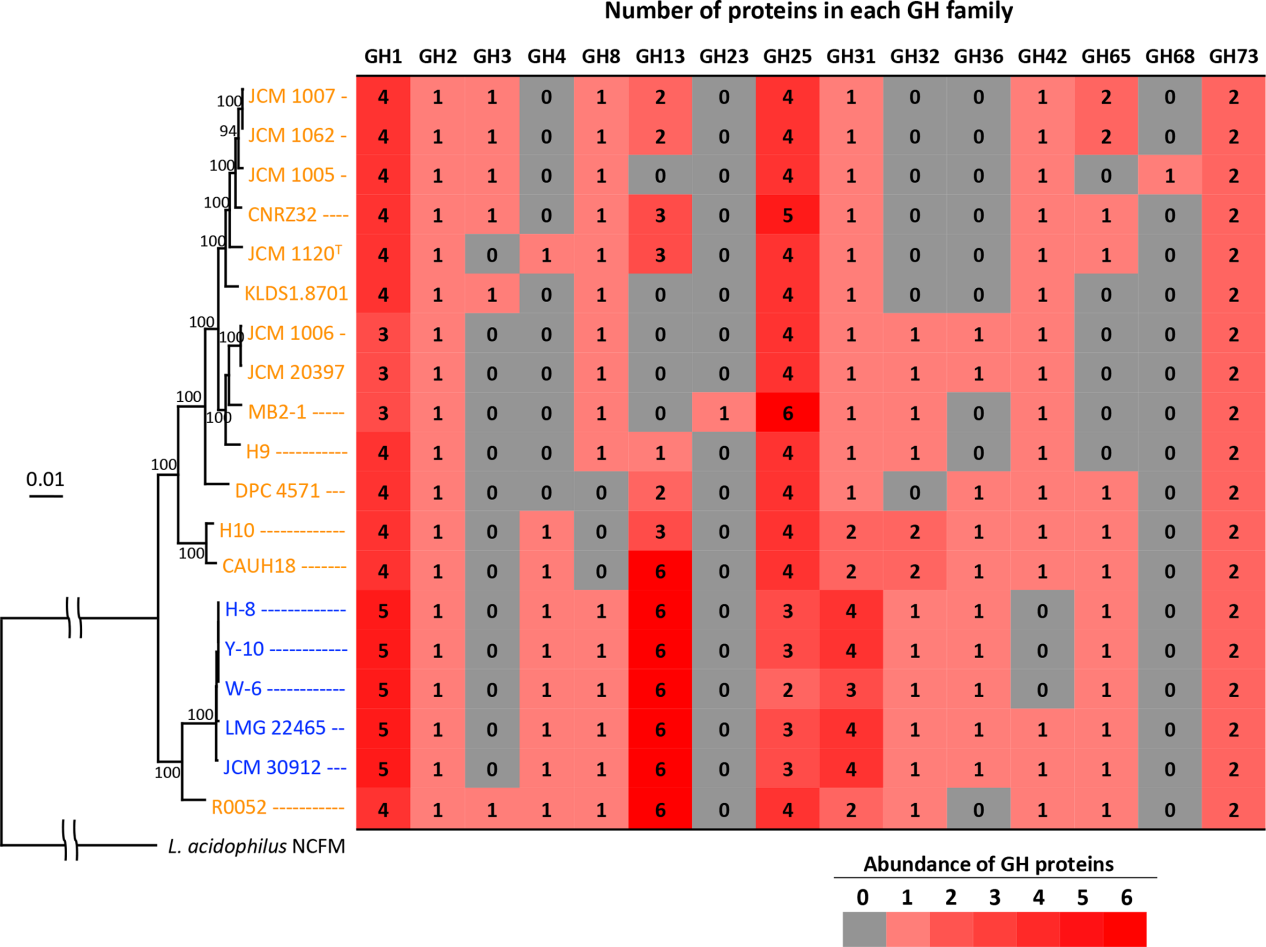
Phylogenetic relationships among 19 strains of *L. helveticus* based on multiple alignments of the 755 conserved genes and number of GH family proteins found in the strains. *L. acidophilus* NCFM was used as an outgroup in the phylogenetic analysis. Bootstrap values over 90 % are indicated in the tree. Strain names of the whisky strains are indicated in blue and those of the milk strains are in orange. The scale bar means substitution per site.

The completeness of the genomic data was assessed by CheckM (version 1.0.11) [26]. This analysis was conducted by including two different gene marker sets for *Lactobacillus* spp. and for *L. helveticus* in the program (Table 1). All versus CRL32 genome alignments were visualized using the blast Ring Image Generator (brig; Fig. S1, available with the online version of this article), including a ring for each genome [27]. blastn was used with the following options: upper identity threshold of 90 % and a lower identity threshold of 70 %. Genetic features with low blast identity were identified through visual genomic inspection. Genome level identities of *L. helveticu *s strains were determined by calculating the average nucleotide identity (ANI), as described previously [28, 29]. ANI values were used to prepare a dendrogram using the hclust function with the Ward.D2 algorithm in the R package (version 3.6.2; Fig. 3). Orthologous clusters that were conserved in 19 strains of *L. helveticus* and *Lactobacillus acidophilus* NCFM (outgroup) were determined using get_homologues software (version 1.3) based on the all-against-all bidirectional blast alignment and the MCL graph-based algorithm [30]. The amino acid sequences of the conserved genes were concatenated and used to reconstruct a phylogenetic tree (Fig. 4), as described previously [20].

**Fig. 5.**
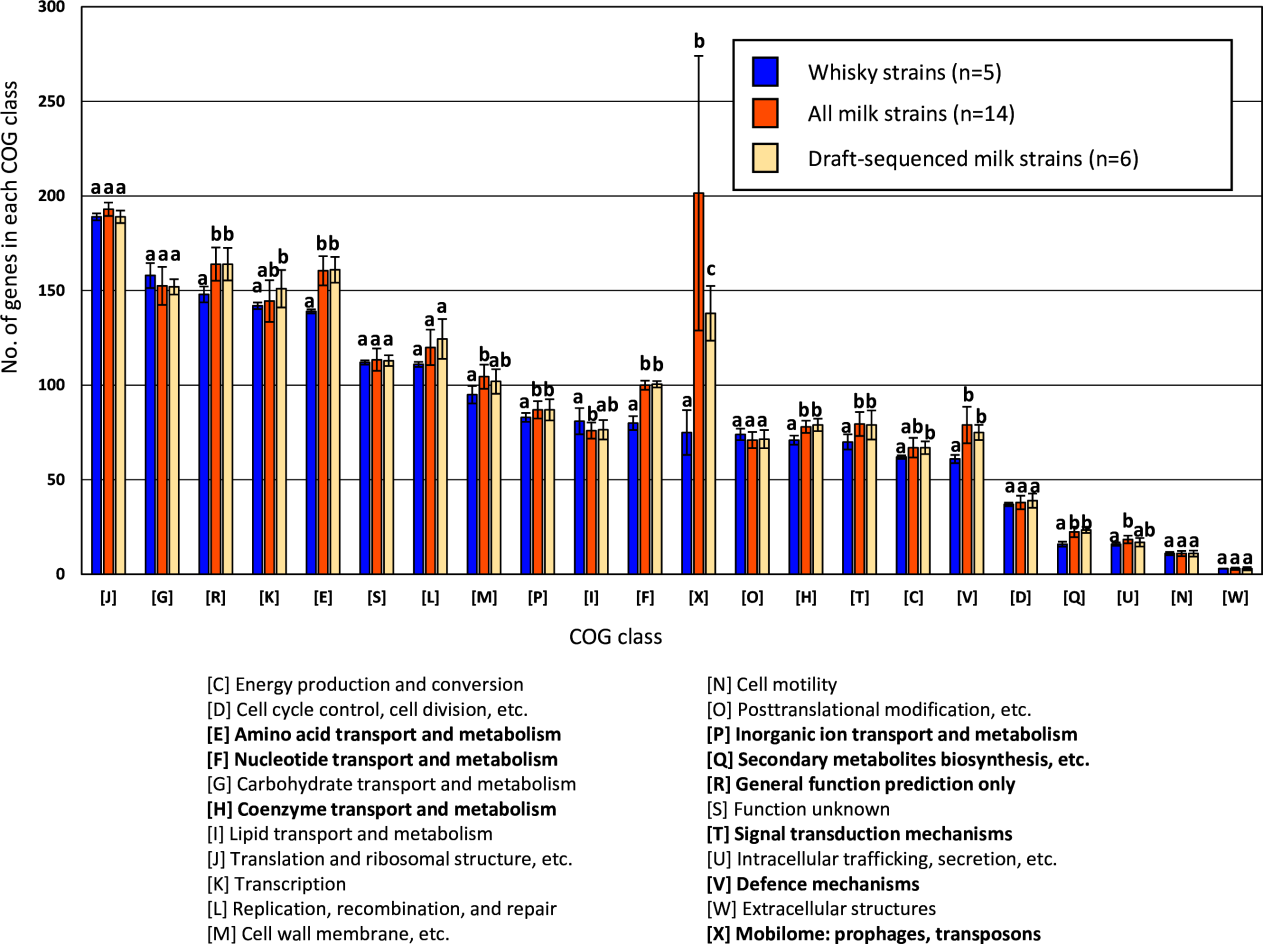
Comparison of gene numbers in each COG class for the whisky strains and milk strains. Bars and error bars indicate means and sd, respectively. The Mann–Whitney *U* test was used to compare between the whisky strains and all milk strains, between the whisky strains and the draft-sequenced milk strains, and between the draft-sequenced milk strains and all milk strains. Different letters on top of the bars indicate significant differences. Class names shown in bold are the classes significantly different between the milk strains and whisky strains.

For functional comparison of the gene contents between milk strains and whisky strains, coding sequences (CDSs) predicted in each strain were assigned to Cluster of Orthologous Groups (COG) functional classification using cognitor software [31] (Fig. 5, Tables S1 and S2). Metabolic pathways in each strain were also predicted using the KEGG Automatic Annotation Server (KAAS) by assigning KEGG Orthology numbers to each predicted CDS [32] (Table S3).

### Analysis of glycoside hydrolase (GH) family proteins

**Fig. 6.**
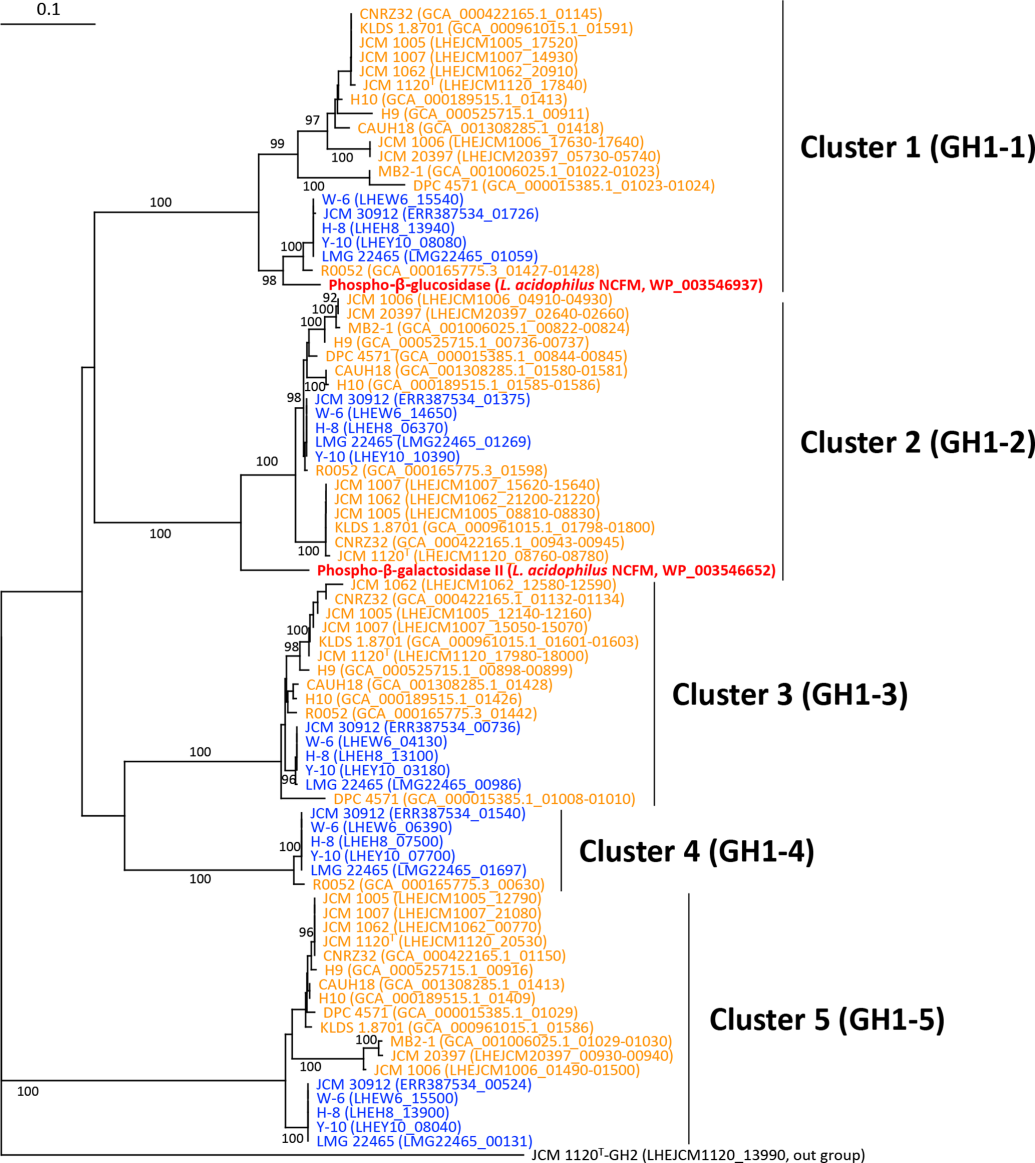
Phylogenetic relationships of GH1 enzymes found in *L. helveticus* strains. Locus tags of proteins and strain names are shown. When ranges of locus tags are shown, amino acid sequences of the genes were concatenated and used for multiple alignments. Strain names of the whisky strains are indicated in blue and those of the milk strains are in orange. Reference GH1 enzymes in *L. acidophilus* NCFM are shown in red. The GH2 enzyme of *L. helveticus* JCM 1120^T^ (LHEJCM1120_13990) was used as an outgroup. Bootstrap percentages above 90% are indicated at branching points. The scale bar means substitution per site.

**Fig. 7.**
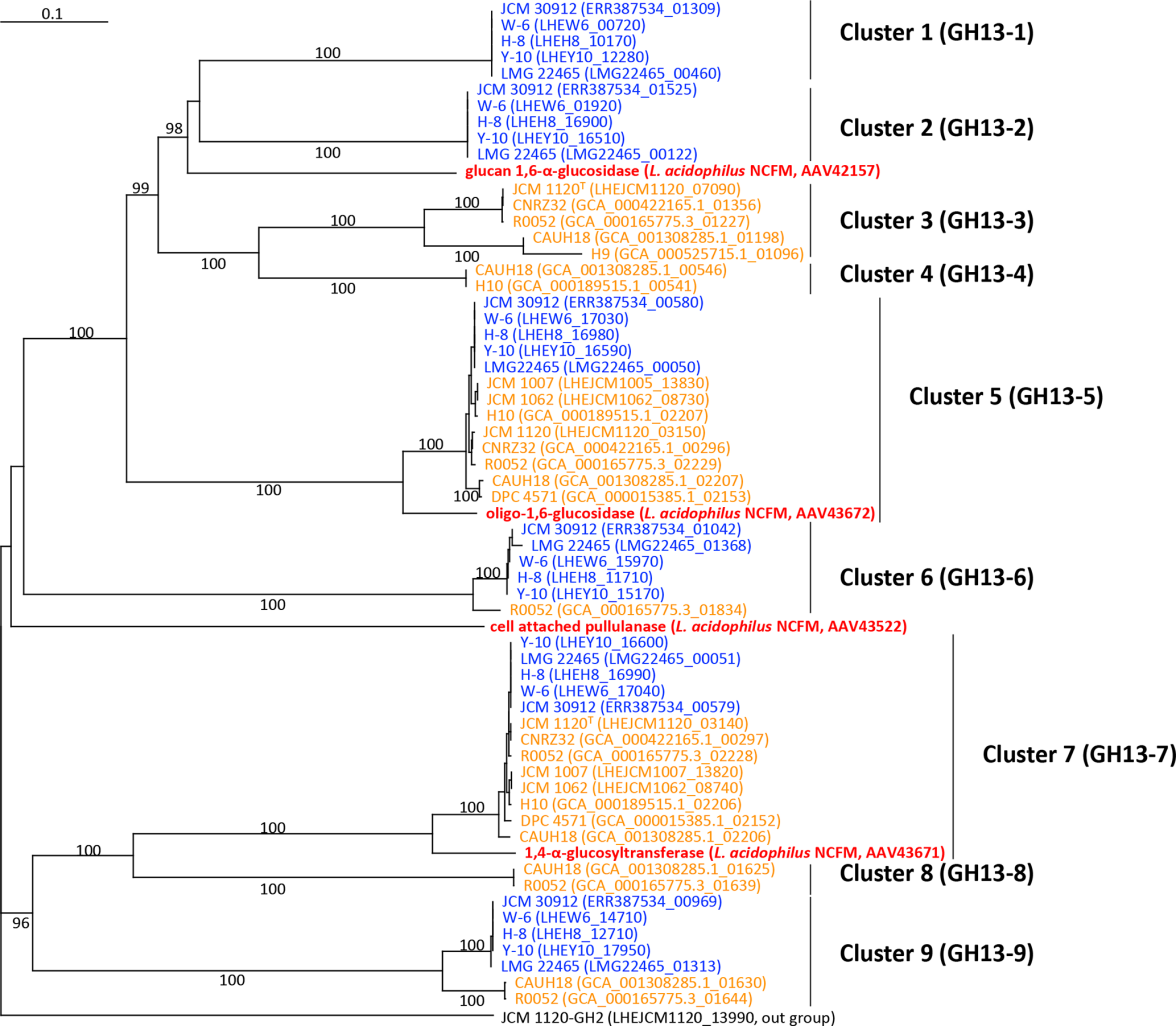
Phylogenetic relationships of GH13 enzymes found in *L. helveticus* strains. Locus tags of the proteins and strain names are shown. Strain names of the whisky strains are indicated in blue and those of the milk strains are in orange. Reference GH13 enzymes in *L. acidophilus* NCFM are shown in red. GH2 enzyme of *L. helveticus* JCM 1120^T^ (LHEJCM1120_13990) was used as an outgroup. Bootstrap percentages above 90% are indicated at branching points. The scale bar means substitution per site.

**Fig. 8.**
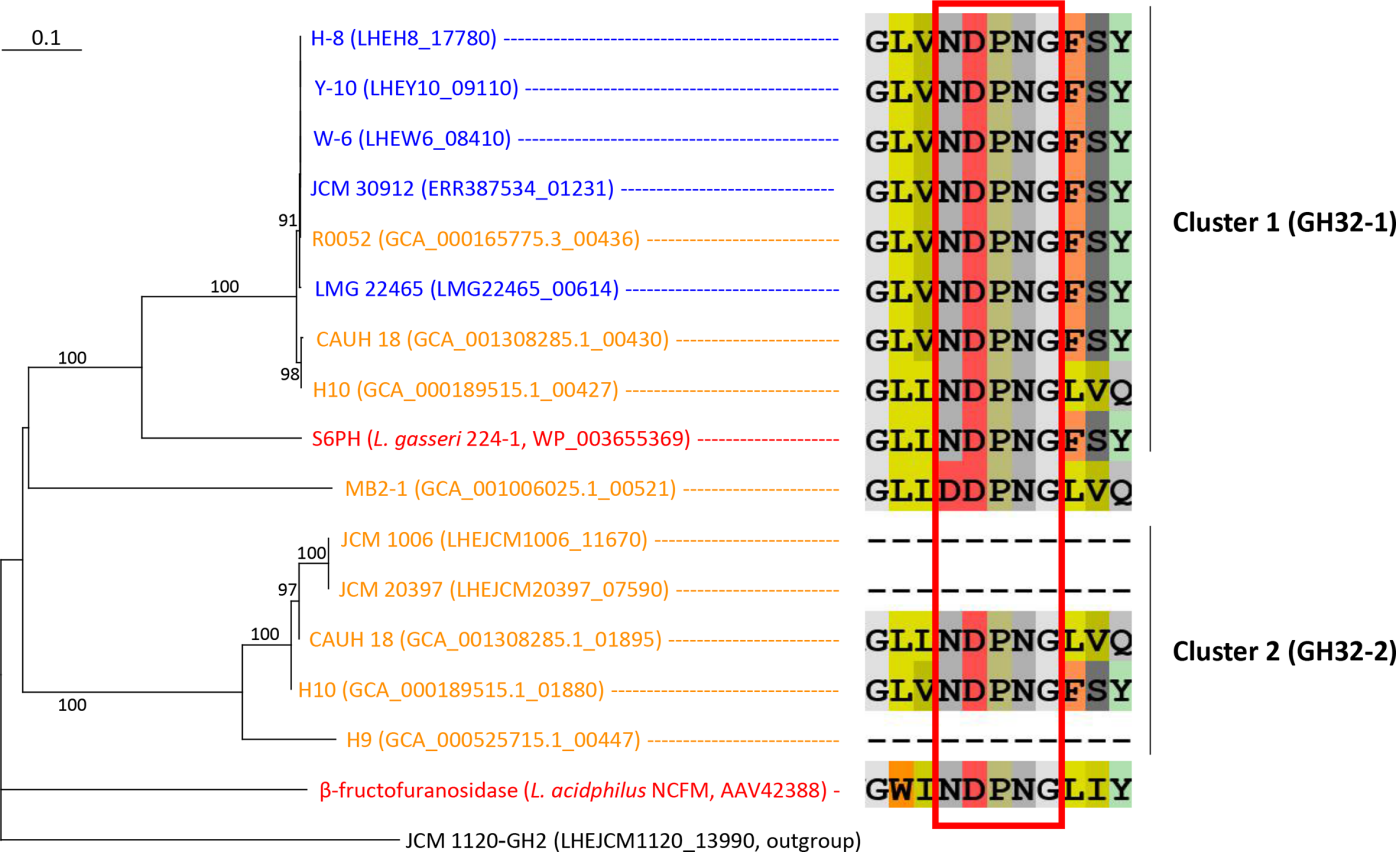
Phylogenetic relationships and sequences around the NDPNG motif of GH32 enzymes found in *L. helveticus* strains. Locus tags of proteins and strain names are shown. Strain names of the whisky strains are indicated in blue and those of the milk strains are in orange. Reference GH32 enzymes, i.e. S6PH in *L. gasseri* 224-1 and β-fructofuranosidase in *L. acidophilus* NCFM, are shown in red. GH2 enzyme of *L. helveticus* JCM 1120^T^ (LHEJCM1120_13990) was used as an outgroup. Bootstrap percentages above 90 % are indicated at branching points. Amino acid sequences around the NDPNG motif (red boxed) region are shown. The scale bar means substitution per site.

The genomic data of 19 strains of *L. helveticus* were used to search for GH family enzymes using dbCAN2 in the CAZy database with hmmer, diamond and Hotpep tools [33]. GH proteins were identified when detected by two of the three tools (Fig. 4), as recommended by the database [33]. blastp analysis was further conducted to search for GH family enzymes manually. The numbers of estimated proteins in each GH family of the strains were used to prepare a dendrogram using the hclust function with the Ward.D2 algorithm in the R package (Fig. S2). The phylogenetic tree was reconstructed for GH enzymes using the program ClustalW, version 2.1 [34] (Figs 6–8 and S3–S6). The number of bootstrapping replicates was 1000.

### Statistical analysis

The Mann–Whitney *U* test was applied to compare stress tolerances between the milk strains and the whisky strains. A *P* value of 0.05 was considered significant. The Mann–Whitney *U* test was also applied to compare genomic features and gene content of the milk strains (*n*=14) and the whisky strains (*n*=5). Moreover, to exclude the bias of different genome status, i.e. completely sequenced and draft-sequenced, between the strains, the Mann–Whitney *U* test was applied to compare between the draft-sequenced milk strains (*n*=6) and the draft-sequenced whisky strains (*n*=5). Statistical analysis was performed using IBM SPSS Statistics for Windows (version 26).

## Results

### Phenotypic characteristics

#### Carbohydrate metabolic properties

All milk strains (*n*=6) actively grew in the presence of galactose and lactose, whereas the whisky strains (*n*=5) exhibited negative reactions with the two milk-origin carbohydrates (Table 2). However, whisky strains had positive reactions with plant-origin carbohydrates, including cellobiose, sucrose, 1-kestose, fructooligosaccharide (FOS) and salicin, whereas none of the milk strains metabolized these carbohydrates. Maltose, a major carbohydrate in malt fermentations, was metabolized by all whisky strains and only one of the six milk strains. The whisky strains also metabolized maltotriose and three of the six milk strains had positive reactions with maltotriose. The three milk strains demonstrated positive or weak reactions with starch, but the remaining three milk strains had negative reactions with maltotriose and starch. All whisky strains exhibited positive reactions with starch.

#### Milk fermentation properties

All milk strains reduced the pH of the UHT milk and skim milk to below 5.1 and coagulated the milks after 48 h of incubation, whereas the pH of the milks remained between 6.3 and 6.5 after fermentation by the whisky strains (Table 3). Coagulation was observed in milks fermented by the milk strains after 24 h of incubation, but not in the milks inoculated by the whisky strains even after 48 h. Supplementation of 1 % glucose in skim milk did not impact the growth abilities of the whisky strains. However, the whisky strains fermented skim milk in the presence of the combination of 1 % glucose and 0.5 % yeast extract. All whisky strains reduced the pH of the skim milk with yeast extract and glucose to below 4.7 and coagulated the milk after 48 h of incubation (Table 3). The addition of casitone did not markedly impact the fermentation of the milk.

#### Stress tolerance

The whisky strains were significantly more tolerant of 7.5 % (v/v) ethanol than the milk strains at 24 h (Fig. 1). Similar tendencies were noted for 2.5 and 5 % ethanol. However, the milk strains were significantly more tolerant of lysozyme (1 and 10 μg ml^−1^) and lactoferrin (1 mg ml^−1^).

### Genome analysis

#### General genomic features of the milk and whisky strains of *L. helveticus*

In the genomic analysis, 8 completely sequenced *L. helveticus* strains originating from milk products were also included in a group of milk strains (Table 1), resulting in 14 milk strains in total. The genome sizes of the whisky strains (*n*=5) were significantly smaller than those of the 14 milk strains (median±sd 1.96±0.09 vs 2.13±0.11 Mbp, *P*=0.004; Fig. 2a). Accordingly, the whisky strains contained significantly fewer CDSs than the milk strains (median±sd 1904±86 vs 2236±119, *P*=0.001; Fig. 2b). Significant differences remained even if the whisky strains and draft-sequenced milk strains were compared (Fig. 2). The completeness of the 19 tested strains ranged from 97–99 % based on the genus *Lactobacillus* core gene markers (Table 1), demonstrating that the genomic data were appropriate for further analyses. When the completeness was determined based on the *L. helveticus* core gene markers, the values for the whisky strains and the milk strains ranged from 89 to 91% and from 96 to 99 %, respectively. The five whisky strains commonly lacked 32 of the 731 marker genes.

The CDSs of *L. helveticus* strains were compared with the reference genome, that of strain CNRZ32. Certain genomic regions were revealed to be CNRZ32-specific regions (blue barred regions in Fig. S1), and they contained genes encoding glycosyl transferases (GTs, mostly GT2 enzymes), prophage-related proteins, cell division-related proteins and hypothetical proteins (Fig. S1). Moreover, several genomic regions were only missing in the whisky strains (red barred regions in Fig. S1), which contained genes encoding transposases, ABC transporters, permeases, proteins involved in metabolism of folate and purine, Na^+^/H^+^ antiporter family protein, glutathione reductase, and hypothetical proteins.

#### DNA-level identities and phylogenetic relationships

The 19 strains of *L. helveticus* (5 whisky strains and 14 milk strains) were separated into two clusters by ANI-value-based clustering (Fig. 3). One cluster contained the five whisky strains and three milk strains, CAUH18, H10 and R0052. The five whisky strains shared ANI values over 0.995 among the strains, but they had relatively lower ANI values (<0.980) with the milk strains, except for the relatively higher ANI values (0.987) with the milk strain R0052. The highest ANI values of strain R0052 were recorded with the whisky strains. Strains CAUH18 and H10 shared ANI values of 0.995, but had relatively low ANI values (<0.982) with the other *L. helveticus* strains. The other cluster included the remaining 11 milk strains, which had high ANI values (>0.987).

Phylogenetic analysis based on multiple alignment of the 755 conserved genes produced two major clusters (Fig. 4). The first cluster contained 13 of the 14 milk strains. The second cluster included the five whisky strains and one milk strain, R0052, while strain R0052 was outside of a robust subcluster composed of the whisky strains.

#### Comparison of gene content based on functional gene categories

The genes identified in *L. helveticus* strains were assigned to COG functional classification, and the number of genes involved in each function was compared between the whisky and milk strains (Table S1). To eliminate biases in genome status (i.e. draft-sequenced and completely sequenced), a group of draft-sequenced milk strains was also included in this comparison. In the analysis, differences were evaluated as significant when *P* values both between the whisky strains and all milk strains, and between the whisky strains and draft-sequenced milk strains, were <0.05. After elimination of the biases, significant differences were found in 9 of the 22 classes (Fig. 5). Of the nine classes, marked differences were found in the number of genes for the mobilome, prophages and transposons (class X), between the milk strains and the whisky strains. The 14 milk strains possess 202±73 (median±sd) genes in this class and this number is significantly higher than that of the whisky strains (median±sd 75±12, *P*=0.001). Sequencing status (complete vs draft) markedly affected gene numbers of class X in the milk strains (median±sd 263±51 vs 138±14, *P*=0.05), but the gene numbers in class X of the draft-sequenced milk strains (*n*=6) were still significantly higher than those of the whisky strains (median±sd 138±14 vs 75±12, *P*=0.006). Comparison of gene content in class X revealed that larger numbers of transposases belonging to COG0675 [insertion sequence 605 (IS*605*) family], COG2826 (IS*30* family), COG3039 (IS*5* family), COG3328 (IS*285* family), COG3385 (IS*4* family), COG3464 (IS*204*, IS*100*, IS*110*, IS*961* and IS*165* families) and COG3666 (IS family unknown) were present in milk strains (Table S2). Classes that significantly differed between the milk strains and the whisky strains included class E amino acid transport and metabolism (median±sd 160.5±8 vs 139±1), and class F nucleotide transport and metabolism (100±2 vs 80±4) (Fig. 5).

The KAAS system was applied to compare genes involved in metabolic/biosynthesis pathways. The whisky strains have a significantly larger number of genes involved in starch and sucrose metabolism (median ±SD 22±0.5 vs 11.5 vs 4.9, *P*=0.005), and the phosphotransferase system (PTS) (median±sd 16±0 vs 10.5 vs 2.5, *P*=0.005), but contain fewer genes involved in purine metabolism (median±sd 36±5.8 vs 47±3.1, *P*=0.002), cysteine and methionine metabolism (median±sd 13±0.4 vs 17±1.3, *P*=0.001), and folate biosynthesis (median±sd 2±0 vs 6±1.7, *P*=0.003) (Table S3). Significant differences remained when the whisky strains and draft-sequenced milk strains were compared (data not shown). All milk strains possess a complete gene set involved in the Leloir pathway for galactose metabolism, but only two (JCM 30912 and LMG 22465) of the five whisky strains possess the complete gene set. The remaining three whisky strains (H-8, W-6 and Y-10) lack aldose 1-epimerase, galactokinase and galactose-1-phosphate-uridyltransferase in the pathway.

#### Identification of GH family proteins

In total, 15 GH families were found in genomes of *L. helveticus* strains (Fig. 4, Table S4). The whisky strains possess significantly larger numbers of GH family proteins than the milk strains (median±sd 26±1.2 vs 18±3.4, *P*=0.002). Proteins assigned to GH4, GH32 and GH36 were conserved in all whisky strains, but were found only in 29, 50 and 36 % of the milk strains, respectively. Hierarchical clustering analysis based on the number of proteins in each GH family produced major two clusters (Fig. S2). One contained 12 of the 14 milk strains, and the other contained the remaining 2 milk strains (strains R0052 and CAUH18) and all the whisky strains. To examine the phylogenetic relationships of proteins assigned to each GH family and to confirm *in vitro* carbohydrate metabolic properties, phylogenetic trees were produced based on the amino acid sequences of GH proteins assigned to GH1, GH4, GH13, GH31, GH32, GH36 and GH65. Well-characterized enzymes involved in the hydrolysis of cellobiose/salicin, maltose, maltooligosaccharides/starch, sucrose/1-kestose/FOS and raffinose in *L. acidophilus* NCFM, and sucrose/1-kestose/FOS in *Lactobacillus gasseri* 224–1 were also included in the phylogenetic analyses as references.

Five and three to four proteins were assigned to GH1 in the whisky strains and milk strains, respectively (Fig. 4). The analysis revealed that several genes encoding GH1 enzymes in the milk strains were divided into two or three genes by the presence of stop codons, whereas these genes were intact in the whisky strains. Amino acid sequences of the divided genes were, therefore, concatenated and used for multiple alignments. Two reference GH1 enzymes in *L. acidophilus* NCFM involved in the metabolism of cellobiose and salicin were also included in the phylogenetic analysis. The phylogenetic tree produced five distinct clusters (Fig. 6) and each whisky strain possessed a single protein belonging to the five clusters. The two references, i.e. phospho-β-glucosidase and phospho-β-galactosidase II, belong to the clusters 1 (GH1-1) and 2 (GH1-2), respectively. The genes encoding GH1-1 and GH1-2 were found in all strains tested, whereas these genes were inactive in the milk strains due to the presence of stop codon(s) and/or partial deletion, except for GH1-2 in strain R0052. This is described in detail in the section ‘Inactivation pattern of GH1-1 and GH1-2 in the milk strains’. Sequence similarities of GH1-1 within the whisky strains were over 99 % and shared approximately 93 % similarity with the reference phospho-β-glucosidase in *L. acidophilus* NCFM. The GH1-2 enzymes in the whisky strains shared approximately 86 % similarity with the reference phospho-β-galactosidase II in *L. acidophilus *. Proteins belonging to cluster 4 were only found in the whisky strains and strain R0052.

A single GH4 protein was conserved in all whisky strains, but was found in only 4 of the 14 milk strains (Fig. 4). Strain JCM 1120^T^, which was the only strain that metabolized maltose among the six tested milk strains (Table 1), possessed GH4 protein. Milk strains CAUH18, H10 and R0052, which produced a cluster with the whisky strains by ANI-value-based clustering (Fig. 3), also possessed a single GH4 protein. Phylogenetic analysis revealed that the GH4 proteins, annotated as maltose-6′-phosphate glucosidase or 6-phospho-α-glucosidase (Table S4), found in *L. helveticus* strains shared over 99 % similarity (Fig. S3).

The numbers of proteins assigned to GH13 greatly differed among the tested strains. Six and zero to six proteins assigned to GH13 were found in the whisky and milk strains, respectively (Fig. 4). The milk strains possessing six GH13 proteins were R0052 and CAUH18. Phylogenetic analysis of the GH13 proteins and four reference GH13 enzymes in *L. acidophilus* NCFM produced nine clusters (Fig. 7). Clusters 1 (GH13-1) and 2 (GH13-2) contained proteins only found in the whisky strains. Cluster 6 (GH13-6) contained proteins from the whisky strains and R0052, and cluster 9 (GH13-9) included an additional strain of CAUH18. However, clusters 3 (GH13-3), 4 (GH13-4) and 8 (GH13-8) were composed of proteins only found in certain milk strains, whereas the strains included differed among the clusters. Oligo-1,6-α-glucosidase and 1,4-α-glucosyltransferase of *L. acidophilus* NCFM, which cooperatively function in the hydrolysis of maltooligosaccharides/starch [35], belong to cluster 5 (GH13-5) and cluster 7 (GH13-7), respectively. GH13-5 and GH13-7 proteins were found in all whisky strains and 8 of the 14 milk strains. The remaining two reference GH13 enzymes, i.e. isomaltooligosaccharide hydrolysing 1,6-α-glucosidase [36] (GenBank accession no. AAV42157) and extracellular cell-attached pullulanase [37] (GenBank accession no. AAV43522), in *L. acidophilus* did not belong to any cluster.

GH65 proteins were also conserved in all whisky strains (*n*=5), whereas the proteins were found in 8 of the 14 milk strains (Fig. 4). Of the eight milk strains, two (JCM 1007 and JCM 1062) possessed two proteins assigned to GH65. Phylogenetic analysis of these GH65 proteins and the reference GH65 maltose phosphorylase in *L. acidophilus* [38] produced two distinct clusters (Fig. S4). Sequence similarities between proteins belonging to the different clusters were below 40 %. All whisky strains and the eight milk strains possessed a single protein in cluster 1 (GH65-1), and this cluster included the reference maltose phosphorylase in *L. acidophilus *, suggesting that GH65-1 proteins in *L. helveticus* are also maltose phosphorylases. Cluster 2 (GH65-2) contained GH65 proteins found in JCM 1007 and JCM 1062, and these enzymes were annotated as trehalose 6-phosphate/kojibiose phosphorylase (Table S4).

Phylogenetic analysis of GH32 proteins produced two phylogenetic groups and phylogenetically distant proteins from MB2-1, and a reference β-fructofuranosidase of *L. acidophilus* [39] (Fig. 8). Cluster 1 (GH32-1) contained a reference enzyme sucrose-6-phosphate hydrolase (S6PH) of *L. gasseri *, possibly involved in the hydrolysis of sucrose, 1-kestose and FOS. These were found in all whisky strains and 3 (strains CAUH18, H10 and R0052) of the 14 milk strains, but not in milk strains exhibiting negative reactions with sucrose, 1-kestose or FOS in the carbohydrate metabolic test. Proteins belonging to cluster 2 (GH32-2) were found in five milk strains. Sequence similarities between the proteins belonging to the different clusters were below 30 %. Sequence coverage against S6PH in *L. gasseri* was 100 % in GH32-1 proteins, but only 14 % in GH32 of MB2-1, and ranged from 57 to 87 % in GH32-2 proteins. Sequence alignment of the GH32 proteins revealed that the NDPNG motif was conserved in the reference enzymes *L. gasseri* S6PH and *L. acidophilus* β-fructofuranosidase, all GH32-1 enzymes, and two of the five GH32-2 enzymes (Fig. 8). The remaining three GH32-2 enzymes lacked the approximately 200 N-terminal amino acid residues containing the NDPNG motif. GH32 in MB2-1 had one amino acid substitution (DDPNG).

A single GH36 protein was found in all whisky strains (*n*=5) and five of the milk strains. Phylogenetic analysis divided the enzymes into two clusters (Fig. S5). Cluster 1 (GH36-1) included a reference MelA α-galactosidase of a raffinose operon in *L. acidophilus* [40] and five enzymes found in the milk strains. The reference MelA and GH36-1 enzymes in *L. helveticus* strains had relatively high similarity (approx. 70 %), but a different length, i.e. 732 amino acid residues in the reference enzyme and only 285 to 435 amino acid residues in *L. helveticus* GH36-1 enzymes, suggesting that the GH36-1 proteins in the milk strains are inactive. GH36 proteins found in the whisky strains belonged to cluster 2 (GH36-2), but no proteins found in the milk strains belonged to this cluster.

GH31 proteins were abundant in the whisky strains and three to four proteins were conserved in these strains, but only one or two were conserved in the milk strains. Phylogenetic analysis of the GH31 proteins produced four clusters (Fig. S6). Cluster 1 (GH31-1) contained single proteins in all tested strains, except the whisky strain W-6. Proteins in clusters 3 (GH31-3) and 4 (GH31-4) were only found in the whisky strains. Cluster 2 (GH31-2) contained single proteins found in all whisky strains and 3 of the 14 milk strains, including CAUH18, H10 and R0052.

#### Polysaccharide utilization locus (PUL)

**Fig. 9.**
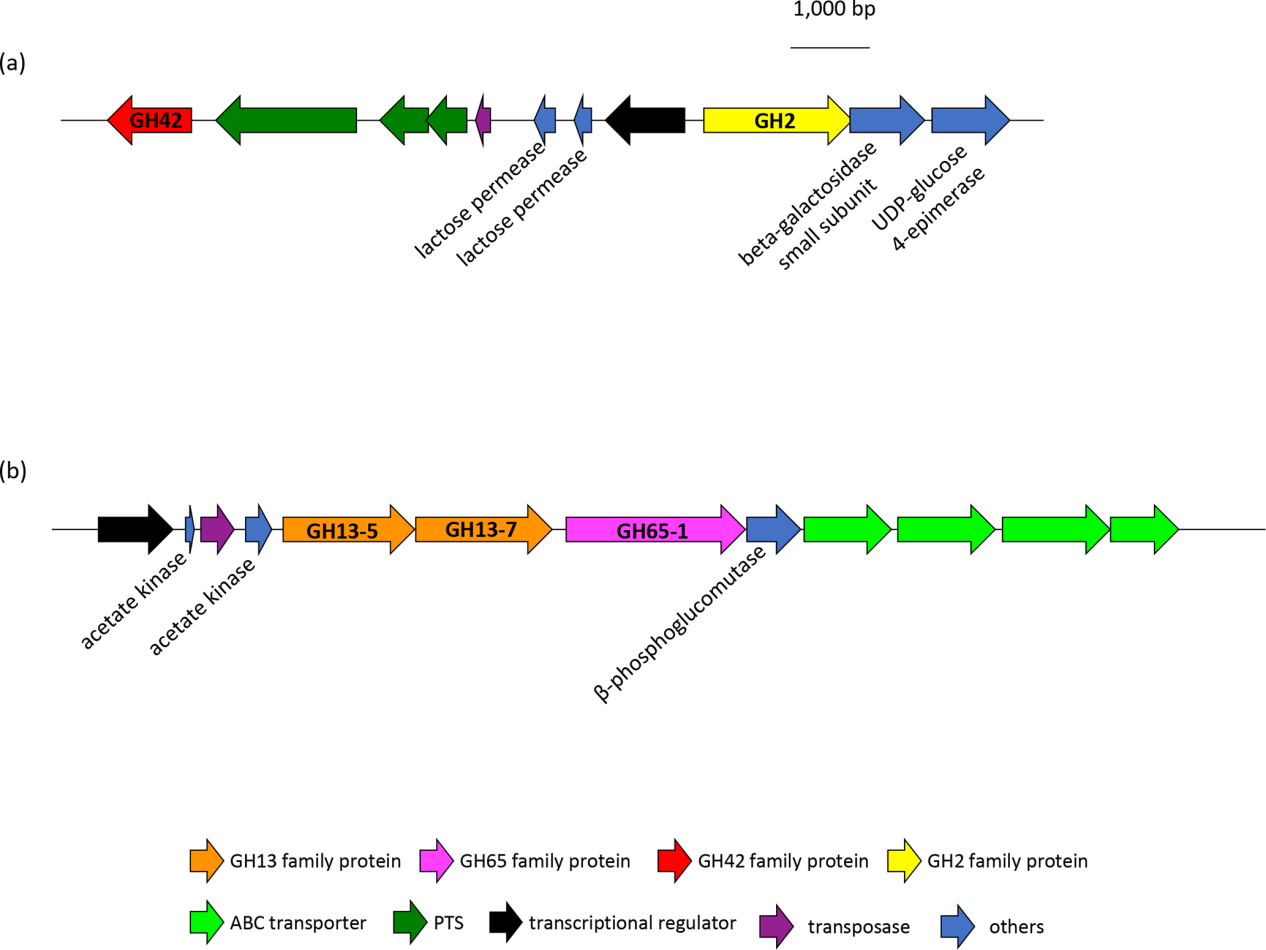
Gene arrangements of PUL-GH2-GH42 (**a**) and PUL-GH13-GH65 (**b**) in CNRZ32. Locus tags of proteins in the PULs range from GCA_000422165.1_01875 to GCA_000422165.1_01885, and from GCA_000422165.1_00292 to GCA_000422165.1_00303, respectively.

A single GH2 enzyme, β-galactosidase large subunit, was conserved in all *L. helveticus* strains tested (Fig. 4). The enzymes shared over 98 % similarity among the strains. The gene encoding GH2 enzyme formed a PUL, as defined elsewhere [41], with genes encoding β-galactosidase small subunit, LacI family transcriptional regulator, lactose permease and UDP-glucose 4-epimerase in all completely sequenced milk strains, and this PUL was adjacent to genes encoding GH42 β-galactosidase and three or four PTS components with/without a transposase, forming a large PUL (PUL-GH2-GH42) containing genes encoding two GH enzymes (Fig. 9a). GH42 β-galactosidase was divided by a stop codon in five (CAUH18, MB2-1, H9, H10 and R0052) of the eight completely sequenced milk strains. This PUL-GH2-GH42 was found in draft-sequenced milk strains with slight modification, although the PUL was divided into two or three contigs in several strains, possibly due to the presence of transposase in this PUL. Genomes of two (JCM 30912 and LMG 22465) of the five whisky strains contained the PUL-GH2-GH42 in divided contigs, whereas those of remaining three (H-8, W-6 and Y-10) lacked lactose permease, LacI family transcriptional regulator, GH42 β-galactosidase and PTS components.

Genes encoding GH13-5 and GH13-7, possibly involved in the hydrolysis of maltooligosaccharides and starch, were adjacent to a gene encoding GH65-1 maltose phosphorylase and produced a single PUL (PUL-GH13-GH65) containing genes encoding a LacI family transcriptional regulator, ABC transporters, acetate kinases and β-phosphoglucomutase in strain CNRZ32 (Fig. 9b). Similar organization of a PUL, containing GH13-5, GH13-7 and GH65-1, was noted in strains carrying these genes in their genomes. Such strains included 8 of the 14 milk strains and all whisky strains, although the PUL was separated into two contigs in a few draft-sequenced strains. The PUL in the whisky strains further contained genes encoding GH13-2 and GH31-3, annotated as oligo-1,6-glucosidase and α-glucosidase, respectively, forming PUL-GH13-GH65-GH31.

#### Inactivation pattern of GH1-1 and GH1-2 in the milk strains

**Fig. 10.**
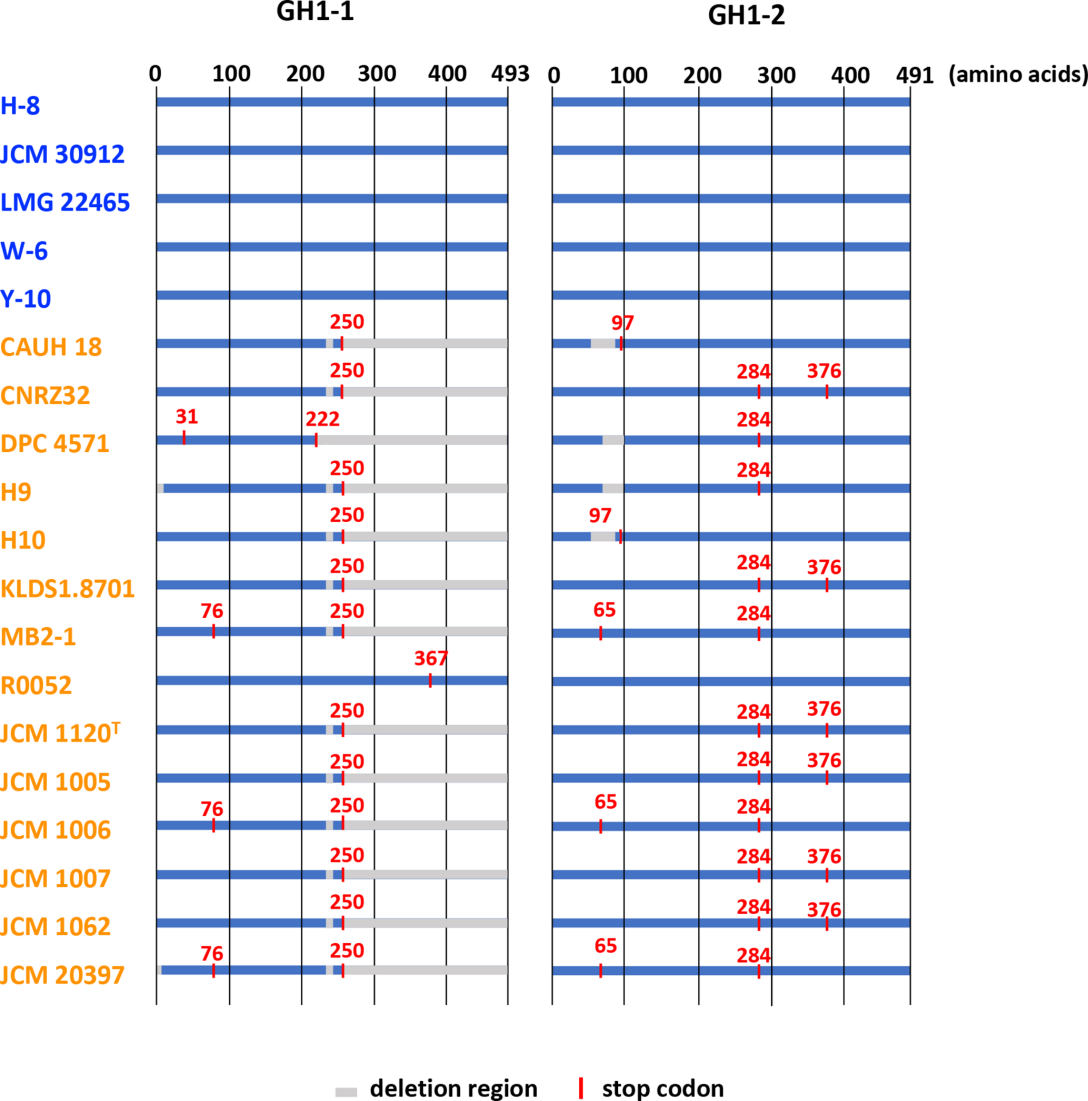
Inactivation patterns of genes encoding GH1-1 (**a**) and GH1-2 (**b**) found in the milk strains. Names of the whisky strains are indicated in blue and those of the milk strains are in orange. Dark blue lines indicate genes in each strain and grey lines indicate deletions of the gene in the region. Red lines on the genes and red numbers indicate positions of stop codons based on protein sequences in the whisky strains. Arrowheads indicate insertion of amino acid sequences (14 residues) that were not found in the protein in the whisky strains. The scale bar means substitution per site.

As described, GH1-1 and GH1-2, consisting of 493 and 491 amino acid residues in whisky strains, respectively, would be key enzymes for the metabolism of cellobiose and salicin, but genes encoding these enzymes were divided or partially deleted by stop codon(s) in the milk strains. The positions of stop codon(s) and partial deletions in GH1-1 and GH1-2 of *L. helveticus* strains after multiple alignments of concatenated sequences are summarized in Fig. 10. A gene encoding GH1-1 was partially deleted after 250 amino acid residues in the N-terminal region (amino acid numbers corresponding to the GH1-1 of JCM 30912) in 12 out of the 14 milk strains. A gene encoding GH1-1 in 3 (MB2-1, JCM 1006 and JCM 20397) of the 12 strains further contained a stop codon at the 76th N-terminal codon. The gene found in DPC 4571 contained a stop codon at the 31st N-terminal codon and lost the sequence after the 222nd amino acid residue, and that in R0052 contained a stop codon at the 367th N-terminal codon without a partial deletion.

The gene encoding GH1-2 was intact in all whisky strains and only 1 (R0052) of the 14 milk strains. GH1-2 in R0052 shares over 99.5 % similarity with those in the whisky strains. The gene encoding GH1-2 in the remaining 13 milk strains was divided into two or three by stop codon(s). The gene encoding GH1-2 in 11 (CNRZ32, DPC 4571, H9, KLDS 1.8701, MB2-1, JCM 1120, JCM 1005, JCM 1006, JCM 1007, JCM 1062 and JCM 20397) of the 13 milk strains contained a stop codon at the 284th N-terminal codon, and those in 9 of the 11 strains further contained a stop codon at the 65th N-terminal codon (MB2-1, JCM 1006 and JCM 20397) or 376th N-terminal codon (CNRZ32, KLDS 1.8701, JCM 1120, JCM 1005, JCM 1007 and JCM 1062). The gene in CAUH18 and H10 contained a stop codon at the 97th N-terminal codon.

## Discussion

The whisky strains demonstrated markedly different phenotypic characteristics from the milk strains. The whisky strains were more tolerant of ethanol (7.5 %) than the milk strains, whereas the milk strains were more tolerant of lysozyme (1 mg ml^−1^) and lactoferrin (10 μg ml^−1^) than the whisky strains. Ethanol is the major inhibitor of microbial growth in alcoholic fermentation, whereas lysozyme and lactoferrin are well characterized growth inhibitors in animal milks [42]. The ethanol concentration of 7.5 % is similar to that at the late stage of malt whisky fermentation and the whisky strains dominate at this stage of fermentation [14]. Carbohydrate metabolic properties also distinguished the *L. helveticus* strains based on their origins. The milk-derived carbohydrates lactose and galactose were only metabolized by the milk strains, whereas several plant-origin carbohydrates, including cellobiose, salicin, sucrose, 1-kestose and FOS, were metabolized only by the whisky strains. Maltose was metabolized by all whisky strains and only one of the six milk strains. Similar distinct carbohydrate metabolic properties of *L. helveticus* strains were reported recently [43]. Of note, although the whisky strains did not ferment skim milk supplemented with glucose, they fermented skim milk supplemented with glucose and yeast extract, suggesting that they need specific growth factor(s) in yeast extract. Yeast extract is usually a source of vitamins, free amino acids, peptides, minerals and nucleotides for culturing lactobacilli. Indeed, whisky strains grow in malt whisky fermentation after alcoholic fermentation and yeast cell autolysis [14]. *L. helveticus* was found from a few cereal fermentations, including sourdoughs and sorghum beers [43, 44], and these fermentations are also conducted in the presence of yeasts. This suggests that *L. helveticus* strains are well adapted to survive in their niches, and the milk-origin- and the whisky-origin-* L. helveticus* are phenotypically distinguished.

Genomic analysis revealed that the whisky strains possess significantly fewer CDSs in their small genomes than the milk strains. This was further confirmed by the genome completeness analysis and brig (blast Ring Image Generator) analysis (Table 1, Fig. S1). Functional gene classification revealed that the genes in class X involved in the IS family transposases were underrepresented in the whisky strains. The presence of the large number of IS family proteins is one of the genomic characteristics of *L. helveticus* strains and this is generally considered to reflect ongoing genome decay [45–47]. Similar genomic features were observed in other dairy lactobacilli [48, 49]. Although the number of genes in class X was influenced by the genome status, i.e. draft sequencing versus complete sequencing, the gene numbers in the whisky strains were only 54 % of those in the draft-sequenced milk strains. As the large number of IS family proteins in class X was suggested to be evidence of ongoing genome decay in *L. helveticus* as described above, the genomes of the whisky strains may be more stable than those of the milk strains. This may be related to less genomic diversity, deduced from higher ANI values, in the whisky strains.

GH profiling was conducted to confirm the marked differences in carbohydrate metabolic properties between the whisky strains and milk strains, and it demonstrated the evolution and adaptation of *L. helveticus* strains to their habitats at the genomic level. Two GH1 enzymes, i.e. phospho-β-glucosidase and phospho-β-galactosidase II, are involved in the hydrolysis of cellobiose and salicin in *L. acidophilus* NCFM. These enzymes belonged to GH1-1 and GH1-2 clusters, respectively, in the phylogenetic analysis, suggesting that GH1-1 and GH1-2 enzymes are involved in the metabolism of cellobiose and salicin in *L. helveticus *. All tested strains had a single gene in each cluster, whereas these genes in the milk strains were divided or partially deleted by stop codon(s). This suggests that GH1-1 and GH1-2 in the milk strains are not functional, except for an intact gene encoding GH1-2 in R0052. R0052 metabolizes cellobiose and salicin, but this is rare in *L. helveticus* [16]. The presence of the incomplete genes is a reason for the deficiency of salicin and cellobiose metabolism in the milk strains.

Different sucrose/1-kestose/FOS metabolic properties were characterized by the presence of GH32-1 proteins. GH32-1 included the reference enzyme S6PH of *L. gasseri* and all GH32-1 enzymes had the conserved NDPNG motif, one of the key regions for the hydrolysis of sucrose/FOS in GH32 enzymes [50, 51]. Previous studies found that a single amino acid substitution in the NDPNG motif (replacement of the initial N to S in the NDPNG motif) significantly reduced the activity of the bacterial GH32 enzyme [52]. The presence of GH32-1 correlated well with the sucrose/1-kestose/FOS metabolic properties of the strains. All whisky strains and three milk strains, CAUH18, H10 and R0052, possessed a single GH32-1, and no milk strains included in the *in vitro* carbohydrate metabolic tests had the enzyme. These implicate that GH32-1 is S6PH and is responsible for the hydrolysis of sucrose/1-kestose/FOS in the whisky strains. GH32-2 enzymes were found in 5 of the 14 milk strains, including JCM 1006 and JCM 20397. Two of the five had the intact NDPNG motif, but the remaining three, including the GH32 enzymes of JCM 1006 and JCM 20397, lacked the approximately 200 N-terminal amino acid residues containing the NDPNG motif when compared with GH32-1. This would result in the deficiency of sucrose/1-kestose/FOS hydrolysis by the enzymes and GH32-2 may be inactive. Based on GH32 distribution, sucrose/1-kestose/FOS metabolism is not common for dairy-origin *L. helveticus* strains.

GH4 maltose-6′-phosphate glucosidase was conserved in all whisky strains and four milk strains, JCM 1120, CAUH18, H10 and R0052. The presence of GH4 maltose-6'-phosphate glucosidase was consistent with the maltose metabolic properties of *L. helveticus* strains used in the *in vitro* carbohydrate metabolic test, and maltose metabolism in R0052 was previously reported [16].

GH13-5 and GH13-7 included reference maltooligosaccharide/starch degrading enzymes of *L. acidophilus* [35]. The genes encoding the two enzymes were located adjacently in the genome and were next to a gene encoding GH65-1 maltose phosphorylase, resulting in the formation of PUL-GH13-GH65 in the milk strain CNRZ32. Similar PUL organization was observed in several species in the *L. acidophilus* group [35]. The PUL was characterized by the release of maltose from maltooligosaccharides through the cooperation of two GH13 enzymes and further phosphorylation of maltose into β-d-glucose 1-phosphate and glucose by the GH65 enzyme in *L. acidophilus *. Similar function is expected for PUL-GH13-GH65 of *L. helveticus *. PUL-GH13-GH65 was conserved in all whisky strains and 7 of the 14 milk strains. When one of the three GHs was conserved in genomes, the genomes contained the PUL-GH13-GH65. The presence of PUL-GH13-GH65 in genomes was consistent with the metabolic properties of maltotriose and starch in *L. helveticus* included in the *in vitro* carbohydrate metabolic test.

PUL-GH2-GH42 containing the lactose operon was conserved in all completely sequenced milk strains. A possible PUL-GH2-GH42 was also found in all draft-sequenced milk strains and two of the whisky strains (JCM 30912 and LMG 22465), although the PUL was separated into two or three contigs. The separation of the PUL may be due to the presence of transposase in the region, as in PUL-GH2-GH42 of CNRZ32 (Fig. 9a). The remaining three whisky strains (H-8, W-6 and Y-10) partially possessed the lactose operon, but lacked lactose permease and a LacI family transcriptional regulator. GH42 β-galactosidase was also not found in these three strains. The role of GH42 in the PUL is unclear, because GH42 usually shares similar activity with GH2 [53]. These two GHs may act in concert to efficiently hydrolyse lactose, but GH42 is not essential because it is present in an inactive form in five of the eight completely sequenced milk strains. The three whisky strains that lacked a partial lactose operon also lacked several enzymes involved in galactose metabolism of the Leloir pathway, suggesting the lack of these key enzymes as a reason for the metabolic deficiency of lactose and galactose in the three strains. However, two whisky strains, JCM 30912 and LMG 22465, contained complete gene sets of the lactose operon and Leloir pathway; therefore, it is not clear why the two strains did not metabolize galactose and lactose. Further studies are needed to resolve this discrepancy.

Of note, genes encoding GH1-1 and GH1-2 enzymes, possibly involved in the hydrolysis of cellobiose and salicin, were divided into two or three or partially deleted in the genomes of all milk strains, with the exception of GH1-2 of strain R0052, as described above. Those genes were intact in the whisky strains. Therefore, intact genes were originally present in *L. helveticus *, and mutagenesis occurred during adaptation to dairy environments in the milk strains due to unessential characteristics in metabolism of the two carbohydrates in their habitats, suggesting a possible origin of *L. helveticus *. Possible IS-associated gene deletion and decay were suggested in *L. helveticus* [45], and multiple transposases are present surrounding the genes encoding GH1-1 in genomes of the completely sequenced strains (data not shown). Positions of the stop codons in GH1-1 and GH1-2 were shared between several milk strains, e.g. the stop codon present at the 250th codon of GH1-1 in 12 of the 14 milk strains, and 284th and 376th codons of GH1-2 in 11 and 6 of the milk strains, respectively. Certain strains completely shared the positions of stop codons in the two genes, which were (i) CAUH18 and H10, (ii) CNRZ32, KLDS1.8701, JCM 1120^T^, JCM 1005, JCM 1007 and JCM 1062, and (iii) MB2-1, JCM 1006 and JCM 20397. DPC4571, H9 and R0052 were distinct from all other strains. This grouping correlated well with clustering based on the core-genome phylogenetic tree (Fig. 4), demonstrating that evolution of the two genes reflects the evolution and diversification of the milk strains.

Strain R0052, originally isolated from sweet acidophilus milk [16], belonged to the cluster mainly consisting of the whisky strains based on core-genome phylogenetic tree and ANI-value-based clustering (Figs 3 and 4). The strain had several GHs that were uncommon in the milk strains but well conserved in the whisky strains, including GH1-4, GH4, GH13-6, GH13-9, GH31-2 and GH32-1. A previous study found unusual carbohydrate metabolic properties in the strain, including the metabolism of cellobiose, salicin, maltose and sucrose [16]. The reported trait correlated well with the GH profiling in the present study. This suggested that strain R0052 is closely related to the whisky strains and may be an intermediate strain during the diversification of *L. helveticus* strains. Inactivation patterns of GH1-1 and GH1-2 in the milk strains support this finding. Strains CAUH18 and H10 also contained several GH proteins, including GH4, GH31-2 and GH32-1, which were common in the whisky strains but rare in the milk strains. ANI-value-based clustering and the core-genome phylogenetic tree revealed their distinct positions between the milk strains and whisky strains, suggesting that they are also intermediates between the two groups, but more related to the milk strains than strain R0052.

### Conclusion

The present study demonstrated that *L. helveticus* strains are phenotypically well adapted to their habitats. The milk strains metabolized milk-origin carbohydrates, whereas the whisky strains metabolized plant-origin carbohydrates. The distinct metabolic properties were supported by the presence of intact GHs in their genomes, confirming that *L. helveticus* strains have genomically adapted well to their habitats. Inactivation of genes encoding GH enzymes was noted in genomes of the milk strains. Inactivation patterns of genes encoding GH1-1 and GH1-2 in milk strains suggested possible evolutional patterns during adaptation to the milk environments. Strains CAUH18, H10 and R0052 are interesting intermediate strains during the diversification of *L. helveticus* strains, whereas R0052 is more related to the whisky strains.

## Supplementary Data

Supplementary material 1
