# Supplementary material for: Niche-specific adaptation of Lactobacillus helveticus strains isolated from malt whisky and dairy fermentations
Source: Microb Genom. 2021 Apr 26;7(4):000560. doi: 10.1099/mgen.0.000560 (PMC8208680; doi:10.1099/mgen.0.000560)

Supplemental Table S1. Gene content profiles obtained for *L. helveticus* strains

	Milk strains											
	Draft-sequenced											
	JCM 1120		JCM 1005		JCM 1006		JCM 1007		JCM 1062		JCM 20397	
	No.*	%**	No.	%	No.	%	No.	%	No.	%	No.	%
[C] Energy production and conversion	63	3.0	67	3.1	67	3.3	71	3.2	71	3.2	64	3.1
[D] Cell cycle control, cell division, chromosome partitioning	38	1.8	40	1.9	37	1.8	45	2.0	45	2.0	37	1.8
[E] Amino acid transport and metabolism	160	7.7	162	7.5	154	7.6	169	7.6	171	7.6	156	7.6
[F] Nucleotide transport and metabolism	102	4.9	97	4.5	101	5.0	100	4.5	100	4.5	101	4.9
[G] Carbohydrate transport and metabolism	151	7.3	149	6.9	151	7.4	158	7.1	159	7.1	153	7.5
[H] Coenzyme transport and metabolism	74	3.6	74	3.4	79	3.9	80	3.6	82	3.7	79	3.9
[I] Lipid transport and metabolism	76	3.7	64	3.0	77	3.8	75	3.4	77	3.4	77	3.8
[J] Translation, ribosomal structure and biogenesis	190	9.1	188	8.8	187	9.2	194	8.7	194	8.6	187	9.2
[K] Transcription	142	6.8	159	7.4	143	7.0	161	7.2	162	7.2	143	7.0
[L] Replication, recombination and repair	125	6.0	124	5.8	107	5.3	130	5.8	127	5.7	106	5.2
[M] Cell wall/membrane/envelope biogenesis	90	4.3	99	4.6	102	5.0	107	4.8	108	4.8	102	5.0
[N] Cell motility	11	0.5	11	0.5	8	0.4	11	0.5	11	0.5	8	0.4
[O] Posttranslational modification, protein turnover, chaperones	67	3.2	75	3.5	68	3.3	77	3.4	77	3.4	68	3.3
[P] Inorganic ion transport and metabolism	83	4.0	88	4.1	86	4.2	95	4.2	96	4.3	84	4.1
[Q] Secondary metabolites biosynthesis, transport and catabolism	24	1.2	22	1.0	22	1.1	25	1.1	26	1.2	23	1.1
[R] General function prediction only	159	7.6	164	7.6	164	8.1	179	8.0	179	8.0	164	8.0
[S] Function unknown	113	5.4	113	5.3	117	5.7	111	5.0	112	5.0	118	5.8
[T] Signal transduction mechanisms	75	3.6	83	3.9	74	3.6	89	4.0	89	4.0	72	3.5
[U] Intracellular trafficking, secretion, and vesicular transport	22	1.1	17	0.8	16	0.8	17	0.8	17	0.8	16	0.8
[V] Defense mechanisms	83	4.0	71	3.3	78	3.8	75	3.4	75	3.3	75	3.7
[W] Extracellular structures	5	0.2	3	0.1	3	0.1	3	0.1	3	0.1	3	0.1
[X] Mobilome: prophages, transposons	134	6.4	142	6.6	125	6.1	156	7.0	158	7.0	126	6.2

	Milk strains															
	Completely-sequenced															
	CAUH18		CNRZ32		DPC 4571		H9		H10		KLDS1.8701		MB2-1		R0052	
	No.	%	No.	%	No.	%	No.	%	No.	%	No.	%	No.	%	No.	%
[C] Energy production and conversion	67	3.0	72	3.0	59	2.7	57	2.8	74	3.3	60	2.7	65	2.8	70	3.1
[D] Cell cycle control, cell division, chromosome partitioning	33	1.5	39	1.6	36	1.6	38	1.9	40	1.8	34	1.5	36	1.5	42	1.8
[E] Amino acid transport and metabolism	156	7.0	168	7.0	154	7.0	142	7.0	161	7.1	161	7.1	151	6.5	162	7.1
[F] Nucleotide transport and metabolism	102	4.6	98	4.1	97	4.4	99	4.9	106	4.7	98	4.3	102	4.4	99	4.3
[G] Carbohydrate transport and metabolism	170	7.6	164	6.8	152	6.9	140	6.9	165	7.3	138	6.1	145	6.2	169	7.4
[H] Coenzyme transport and metabolism	73	3.3	81	3.4	72	3.3	76	3.8	77	3.4	80	3.5	80	3.4	76	3.3
[I] Lipid transport and metabolism	71	3.2	77	3.2	74	3.4	68	3.4	75	3.3	76	3.4	80	3.4	78	3.4
[J] Translation, ribosomal structure and biogenesis	196	8.8	194	8.1	193	8.8	190	9.4	199	8.8	193	8.6	193	8.3	195	8.5
[K] Transcription	148	6.6	146	6.1	138	6.3	127	6.3	162	7.2	138	6.1	154	6.6	136	5.9
[L] Replication, recombination and repair	125	5.6	127	5.3	108	4.9	104	5.2	123	5.4	110	4.9	117	5.0	111	4.9
[M] Cell wall/membrane/envelope biogenesis	117	5.2	102	4.2	104	4.7	100	5.0	109	4.8	105	4.7	111	4.7	108	4.7
[N] Cell motility	11	0.5	12	0.5	9	0.4	11	0.5	10	0.4	12	0.5	9	0.4	12	0.5
[O] Posttranslational modification, protein turnover, chaperones	70	3.1	74	3.1	68	3.1	62	3.1	74	3.3	70	3.1	72	3.1	72	3.1
[P] Inorganic ion transport and metabolism	86	3.8	89	3.7	84	3.8	79	3.9	85	3.8	89	3.9	88	3.8	91	4.0
[Q] Secondary metabolites biosynthesis, transport and catabolism	19	0.9	25	1.0	19	0.9	23	1.1	21	0.9	25	1.1	19	0.8	17	0.7
[R] General function prediction only	176	7.9	164	6.8	162	7.4	150	7.4	180	7.9	160	7.1	170	7.3	167	7.3
[S] Function unknown	121	5.4	114	4.7	107	4.9	109	5.4	125	5.5	112	5.0	122	5.2	126	5.5
[T] Signal transduction mechanisms	75	3.4	85	3.5	78	3.5	69	3.4	82	3.6	84	3.7	74	3.2	81	3.5
[U] Intracellular trafficking, secretion, and vesicular transport	21	0.9	18	0.7	21	1.0	19	0.9	19	0.8	21	0.9	17	0.7	19	0.8
[V] Defense mechanisms	92	4.1	76	3.2	96	4.4	76	3.8	96	4.2	99	4.4	91	3.9	80	3.5
[W] Extracellular structures	3	0.1	3	0.1	4	0.2	3	0.1	3	0.1	4	0.2	3	0.1	3	0.1
[X] Mobilome: prophages, transposons	217	9.7	336	14.0	300	13.6	203	10.1	200	8.8	302	13.4	262	11.2	264	11.5

	Whisky strains									
	Draft-sequenced									
	H-8		JCM 30912		LMG 22465		W-6		Y-10	
	No.	%	No.	%	No.	%	No.	%	No.	%
[C] Energy production and conversion	62	3.2	63	3.5	64	3.4	62	3.3	62	3.2
[D] Cell cycle control, cell division, chromosome partitioning	37	1.9	39	2.1	38	2.0	37	1.9	37	1.9
[E] Amino acid transport and metabolism	137	7.1	137	7.5	139	7.3	139	7.3	139	7.3
[F] Nucleotide transport and metabolism	80	4.2	77	4.2	72	3.8	80	4.2	81	4.2
[G] Carbohydrate transport and metabolism	158	8.2	163	8.9	164	8.6	149	7.8	152	7.9
[H] Coenzyme transport and metabolism	71	3.7	66	3.6	71	3.7	71	3.7	72	3.8
[I] Lipid transport and metabolism	81	4.2	66	3.6	82	4.3	81	4.3	82	4.3
[J] Translation, ribosomal structure and biogenesis	189	9.8	192	10.5	187	9.8	189	9.9	190	9.9
[K] Transcription	142	7.4	138	7.6	141	7.4	142	7.5	142	7.4
[L] Replication, recombination and repair	112	5.8	109	6.0	110	5.8	112	5.9	111	5.8
[M] Cell wall/membrane/envelope biogenesis	95	4.9	95	5.2	105	5.5	94	4.9	95	5.0
[N] Cell motility	11	0.6	10	0.5	12	0.6	10	0.5	11	0.6
[O] Posttranslational modification, protein turnover, chaperones	75	3.9	71	3.9	68	3.6	75	3.9	74	3.9
[P] Inorganic ion transport and metabolism	83	4.3	78	4.3	84	4.4	82	4.3	83	4.3
[Q] Secondary metabolites biosynthesis, transport and catabolism	16	0.8	13	0.7	15	0.8	16	0.8	16	0.8
[R] General function prediction only	150	7.8	141	7.7	152	8.0	148	7.8	147	7.7
[S] Function unknown	112	5.8	110	6.0	113	5.9	112	5.9	112	5.8
[T] Signal transduction mechanisms	70	3.6	70	3.8	79	4.1	70	3.7	70	3.7
[U] Intracellular trafficking, secretion, and vesicular transport	16	0.8	18	1.0	16	0.8	16	0.8	16	0.8
[V] Defense mechanisms	61	3.2	61	3.3	66	3.5	62	3.3	61	3.2
[W] Extracellular structures	3	0.2	3	0.2	3	0.2	3	0.2	3	0.2
[X] Mobilome: prophages, transposons	80	4.2	52	2.8	69	3.6	75	3.9	81	4.2

* Number of genes assigned to the class

** Ratio of genes assigned to the class against all CDSs

Supplemental Table S2. List of class X COG numbers with marked differences between the whisky strains and the milk strains

		COG675	COG2826	COG3039	COG3328	COG3385	COG3464	COG3666
	IS family	605	30	5	285	4	204, 100, 110, 961, 165	-
		Transposase	Transposase and inactivated derivatives, IS30 family	Transposase and inactivated derivatives, IS5 family	Transposase (or an inactivated derivative)	IS4 transposase	Transposase	Transposase
milk strains	JCM 1120[T]	56*	31	28	32	7	49	21
	JCM 1005	40	23	11	34	5	45	6
	JCM 1006	49	24	35	27	6	42	17
	JCM 1007	59	22	17	41	10	57	15
	JCM 1062	54	23	13	6	8	43	5
	JCM 20397	42	16	12	24	5	47	3
	CAUH18	57	28	19	16	9	34	8
	CNRZ32	45	19	21	52	8	55	2
	DPC 4571	13	21	8	18	11	20	8
	H9	22	16	10	9	3	24	8
	H10	23	11	13	21	13	23	6
	KLDS1.8701	24	15	13	18	13	24	7
	MB2-1	16	22	7	9	7	24	4
	R0052	26	14	11	10	6	22	6
whisky strains	H-8	2	4	1	1	3	10	0
	JCM 30912	9	5	6	4	6	11	0
	LMG 22465	9	6	9	6	6	11	0
	W-6	10	6	7	5	6	13	0
	Y-10	16	6	3	1	3	11	0

*Number of genes assigned to each class

Supplemental Table S3. Number of genes in discriminative pathways between the whisky strains and milk strains

Pathways	Whisky strains	Milk strains	p
Starch and sucrose metabolism (map00500)	22 (0.5)*	11.5 (4.9)	0.005
Prine metabolism (map00230)	36 (5.8)	47 (3.1)	0.002
Cysteine and methionine metabolism (map00270)	13 (0.4)	17 (1.3)	0.001
Folate biosynthesis (map00790)	2 (0.0)	6 (1.7)	0.003
Phosphotransferase system (PTS) (map02060)	16 (0.0)	10.5 (2.5)	0.005

* median (SD)

Supplemental Table S4. List of GH family proteins in *L. helveticus* strains

	JCM 1120		
Locus tag*	Annotated as	Signal peptide	GH
LHEJCM1120_17840	hypothetical protein	N	1-1
LHEJCM1120_08760-08780	hypothetical protein	N	1-2
LHEJCM1120_17980-18000	hypothetical protein	N	1-3
LHEJCM1120_20530	beta-glucosidase	N	1-5
LHEJCM1120_13990	beta-galactosidase large subunit	N	2
LHEJCM1120_19220	maltose-6'-phosphate glucosidase	N	4
LHEJCM1120_04680	hypothetical protein	N	8
LHEJCM1120_07090	alpha,alpha-phosphotrehalase	N	13-3
LHEJCM1120_03150	oligo-1,6-glucosidase	N	13-5
LHEJCM1120_03140	alpha-glycosidase	N	13-7
LHEJCM1120_14560	lysin	Y (1-26)	25
LHEJCM1120_15160	lysin	Y (1-36)	25
LHEJCM1120_19110	hypothetical protein	N	25
LHEJCM1120_06310	lysozeme	N	25
LHEJCM1120_12580	alpha-glucosidase	N	31-1
LHEJCM1120_14060	hypothetical protein	N	42
LHEJCM1120_03130	maltose phosphorylase	N	65-1
LHEJCM1120_04460	N-acetylmuramidase	Y (1-23)	73
LHEJCM1120_15530	N-acetylmuramidase	N	73

*Locus tags to address proteins in genome of the strain JCM 1120^T (BLYW01000001)

Supplemental Table S4. continued.

	JCM 1005		
Locus tag*	Annotated as	Signal peptide	GH
LHEJCM1005_17520	hypothetical protein	N	1-1
LHEJCM1005_08810-08830	hypothetical protein	N	1-2
LHEJCM1005_12140-12160	hypothetical protein	N	1-3
LHEJCM1005_12790	6-phospho-beta-glucosidase	N	1-5
LHEJCM1005_07840	beta-galactosidase	N	2
LHEJCM1005_08520	beta-N-acetylhexosaminidase	N	3
LHEJCM1005_03540	hypothetical protein	N	8
LHEJCM1005_15120	lysin	Y (1-26)	25
LHEJCM1005_18970	lysin	Y (1-36)	25
LHEJCM1005_04870	hypothetical protein	N	25
LHEJCM1005_05410	lysozyme	N	25
LHEJCM1005_09070	alpha-glucosidase	N	31-1
LHEJCM1005_11940	beta-galactosidase	N	42
LHEJCM1005_17070	hypothetical protein	N	68
LHEJCM1005_06920	N-acetylmuramidase	N	73
LHEJCM1005_07960	N-acetylmuramidase	Y (1-23)	73

*Locus tags to address proteins in genome of the strain JCM 1005 (BLYS01000001)

Supplemental Table S4. continued.

	JCM 1006		
Locus tag*	Annotated as	Signal peptide	GH
LHEJCM1006_17630-17640**	hypothetical protein	N	1-1
LHEJCM1006_04910-04930	hypothetical protein	N	1-2
LHEJCM1006_01490-01500	hypothetical protein	N	1-5
LHEJCM1006_11300	beta-galactosidase large subunit	N	2
LHEJCM1006_18400	hypothetical protein	N	8
LHEJCM1006_15780	lysin	Y (1-26)	25
LHEJCM1006_09740	lysin	Y (1-36)	25
LHEJCM1006_08430	hypothetical protein	N	25
LHEJCM1006_01860	lysozyme	N	25
LHEJCM1006_00650	alpha-glucosidase	N	31-1
LHEJCM1006_11670	hypothetical protein	N	32-2
LHEJCM1006_11610	hypothetical protein	N	36-1
LHEJCM1006_03110**	hypothetical protein	N	42
LHEJCM1006_00310	N-acetylmuramidase	Y (1-23)	73
LHEJCM1006_14090	N-acetylmuramidase	N	73

*Locus tags to address proteins in genome of the strain JCM 1006 (BLYT01000001)

**Assigned to GH by manual BLASTp analysis

Supplemental Table S4. continued.

	JCM 1007		
Locus tag*	Annotated as	Signal peptide	GH
LHEJCM1007_14930	hypothetical protein	N	1-1
LHEJCM1007_15620-15640	hypothetical protein	N	1-2
LHEJCM1007_15050-15070	hypothetical protein	N	1-3
LHEJCM1007_21080	6-phospho-beta-glucosidase	N	1-5
LHEJCM1007_10110	beta-galactosidase	N	2
LHEJCM1007_19630	beta-N-acetylhexosaminidase	N	3
LHEJCM1007_14970	hypothetical protein	N	8
LHEJCM1007_13830	alpha-glucosidase	N	13-5
LHEJCM1007_13820	alpha-amylase	N	13-7
LHEJCM1007_10730	lysin	Y (1-26)	25
LHEJCM1007_15960	lysin	Y (1-36)	25
LHEJCM1007_08670	hypothetical protein	N	25
LHEJCM1007_02110	lysozyme	N	25
LHEJCM1007_03290	alpha-glucosidase	N	31-1
LHEJCM1007_05410	beta-galactosidase	N	42
LHEJCM1007_13810	maltose phosphorylase	N	65-1
LHEJCM1007_05370	trehalose 6-phosphate phosphorylase	N	65-2
LHEJCM1007_08190	N-acetylmuramidase	Y (1-23)	73
LHEJCM1007_10440	N-acetylmuramidase	N	73

*Locus tags to address proteins in genome of the strain JCM 1007 (BLYU01000001)

Supplemental Table S4. continued.

	JCM 1062		
Locus tag*	Annotated as	Signal peptide	GH
LHEJCM1062_20910	hypothetical protein	N	1-1
LHEJCM1062_21200-21220	hypothetical protein	N	1-2
LHEJCM1062_12580-12590	hypothetical protein	N	1-3
LHEJCM1062_00770	beta-glucosidase	N	1-5
LHEJCM1062_00490	beta-galactosidase large subunit	N	2
LHEJCM1062_03530	hypothetical protein	N	3
LHEJCM1062_12350	hypothetical protein	N	8
LHEJCM1062_08730	oligo-1,6-glucosidase	N	13-5
LHEJCM1062_08740	alpha-glycosidase	N	13-7
LHEJCM1062_07920	lysin	Y (1-26)	25
LHEJCM1062_21920	lysin	Y (1-36)	25
LHEJCM1062_15400	hypothetical protein	N	25
LHEJCM1062_06260	lysozyme	N	25
LHEJCM1062_02270	alpha-glucosidase	N	31-1
LHEJCM1062_11130	phosphoribosylaminoimidazole-succinocarboxamide synthase	N	42
LHEJCM1062_08750	maltose phosphorylase	N	65-1
LHEJCM1062_18050	kojibiose phosphorylase	N	65-2
LHEJCM1062_07740	N-acetylmuramidase	Y (1-23)	73
LHEJCM1062_15050	N-acetylmuramidase	N	73

*Locus tags to address proteins in genome of the strain JCM 1062 (BLYV01000001)

Supplemental Table S4. continued.

	JCM 20397		
Locus tag*	Annotated as	Signal peptide	GH
LHEJCM20397_05730-05740**	hypothetical protein	N	1-1
LHEJCM20397_02640-02660	phospho-beta-galactosidase II	N	1-2
LHEJCM20397_00930-00940	hypothetical protein	N	1-5
LHEJCM20397_03040	beta-galactosidase	N	2
LHEJCM20397_01470	hypothetical protein	N	8
LHEJCM20397_04650	lysin	Y (1-26)	25
LHEJCM20397_19450	lysin	Y (1-36)	25
LHEJCM20397_15290	hypothetical protein	N	25
LHEJCM20397_14130	lysozyme	N	25
LHEJCM20397_06040	alpha-glucosidase	N	31-1
LHEJCM20397_07590	hypothetical protein	N	32-2
LHEJCM20397_07530	hypothetical protein	N	36-1
LHEJCM20397_03120**	hypothetical protein	N	42
LHEJCM20397_00310	N-acetylmuramidase	Y (1-23)	73
LHEJCM20397_12830	N-acetylmuramidase	N	73

[a]Locus tags to address proteins in genome of the strain JCM 20397 (BLYX01000001)

**Assigned to GH by manual BLASTp analysis

Supplemental Table S4. continued.

	CAUH18		
Locus tag*	Annotated as	Signal peptide	GH
GCA001308285.1_01418	6-phospho-beta-glucosidase	N	1-1
GCA001308285.1_01580-01581	6-phospho-beta-glucosidase	N	1-2
GCA001308285.1_01428	6-phospho-beta-glucosidase	N	1-3
GCA001308285.1_01413	6-phospho-beta-glucosidase	N	1-5
GCA001308285.1_00718	beta-galactosidase	N	2
GCA001308285.1_01995	6-phospho-alpha-glucosidase	N	4
GCA001308285.1_01198	alpha, alpha-phosphotrehalase	N	13-3
GCA001308285.1_00546	alpha, alpha-phosphotrehalase	N	13-4
GCA001308285.1_02207	alpha-glucosidase	N	13-5
GCA001308285.1_02206	alpha-amylase	N	13-7
GCA001308285.1_01625	amylopullulanase	N	13-8
GCA001308285.1_01630	glycogen branching enzyme	N	13-9
GCA001308285.1_00862	lysin	Y (1-26)	25
GCA001308285.1_02247	lysin	Y (1-36)	25
GCA001308285.1_01046	lysin	N	25
GCA001308285.1_01712	lysozyme	N	25
GCA001308285.1_00169	alpha-glucosidase	N	31-1
GCA001308285.1_02091	alpha-glucosidase	N	31-2
GCA001308285.1_00430	sucrose-6-phosphate hydrolase	N	32-1
GCA001308285.1_01895	sucrose-6-phosphate hydrolase	N	32-2
GCA001308285.1_01890	alpha-galactosidase	N	36-1
GCA001308285.1_00724*	beta-galactosidase	N	42
GCA001308285.1_02205	maltose phosphorylase	N	65-1
GCA001308285.1_00202	N-acetylmuramidase	Y (1-23)	73
GCA001308285.1_01817	N-acetylmuramidase	N	73

*Locus tags to address proteins in genome of the strain CAUH18 (GCA_001308285.1)

**Assigned to GH by manual BLASTp analysis

Supplemental Table S4. continued.

	CNRZ32		
Locus tag*	Annotated as	Signal peptide	GH
GCA000422165.1_01145	6-phospho-beta-glucosidase	N	1-1
GCA000422165.1_00943-00945	6-phospho-beta-glucosidase	N	1-2
GCA000422165.1_01132-01134	6-phospho-beta-glucosidase	N	1-3
GCA000422165.1_01150	6-phospho-beta-glucosidase	N	1-5
GCA000422165.1_01883	beta-galactosidase	N	2
GCA000422165.1_02175	beta-N-acetylhexosaminidase	N	3
GCA000422165.1_00155	beta-glucanase	N	8
GCA000422165.1_01356	alpha, alpha-phosphotrehalase	N	13-3
GCA000422165.1_00296	alpha-glucosidase	N	13-5
GCA000422165.1_00297	alpha-amylase	N	13-7
GCA000422165.1_01542	lysin	N	25
GCA000422165.1_01743	lysin	Y (1-26)	25
GCA000422165.1_00245	lysin	Y (1-36)	25
GCA000422165.1_01476	arsenate reductase	N	25
GCA000422165.1_00740	lysozyme	N	25
GCA000422165.1_00191	alpha-glucosidase	N	31-1
GCA000422165.1_01875	beta-galactosidase	N	42
GCA000422165.1_00298	maltose phosphorylase	N	65-1
GCA000422165.1_00221	N-acetylmuramidase	Y (1-23)	73
GCA000422165.1_00692	N-acetylmuramidase	N	73

*Locus tags to address proteins in genome of the strain CNRZ32 (GCA_000422165.1)

Supplemental Table S4. continued.

	DPC 4571		
Locus tag[*]	Annotated as	Signal peptide	GH
GCA000015385.1_01023-01024	6-phospho-beta-glucosidase	N	1-1
GCA000015385.1_00844-00845	phospho-beta-galactosidase II	N	1-2
GCA000015385.1_01008-01010	6-phospho-beta-glucosidase	N	1-3
GCA000015385.1_01029	6-phospho-beta-glucosidase	N	1-5
GCA000015385.1_01670	beta-galactosidase	N	2
GCA000015385.1_02153	alpha-glucosidase	N	13-5
GCA000015385.1_02152	alpha-amylase	N	13-7
GCA000015385.1_01548	lysin	Y (1-26)	25
GCA000015385.1_02208	lysin	Y (1-36)	25
GCA000015385.1_01358	lysin	N	25
GCA000015385.1_00716	lysozyme	N	25
GCA000015385.1_00169	alpha-glucosidase	N	31-1
GCA000015385.1_00534	alpha-galactosidase	N	36-1
GCA000015385.1_01661	beta-galactosidase	N	42
GCA000015385.1_02151	maltose phosphorylase	N	65-1
GCA000015385.1_00209	N-acetylmuramidase	Y (1-23)	73
GCA000015385.1_00611	N-acetylmuramidase	N	73

[*]Locus tags to address proteins in genome of the strain DPC4571 (GCA_000015385.1)

Supplemental Table S4. continued.

	H9		
Locus tag[*]	Annotated as	Signal peptide	GH
GCA000525715.1_00911	6-phospho-beta-glucosidase	N	1-1
GCA000525715.1_00736-00737	phospho-beta-galactosidase II	N	1-2
GCA000525715.1_00898-00899	6-phospho-beta-glucosidase	N	1-3
GCA000525715.1_00916	6-phospho-beta-glucosidase	N	1-5
GCA000525715.1_01528	beta-galactosidase	N	2
GCA000525715.1_00117	beta-glucanase	N	8
GCA000525715.1_01096	alpha, alpha-phosphotrehalase	N	13-3
GCA000525715.1_01386	lysin	Y (1-26)	25
GCA000525715.1_02022	lysin	Y (1-36)	25
GCA000525715.1_01194	lysin	N	25
GCA000525715.1_00624	lysozyme	N	25
GCA000525715.1_00151	alpha-glucosidase	N	31-1
GCA000525715.1_00447	sucrose-6-phosphate hydrolase	N	32-2
GCA000525715.1_01520[**]	beta-galactosidase	N	42
GCA000525715.1_00183	N-acetylmuramidase	Y (1-23)	73
GCA000525715.1_00528	N-acetylmuramidase	N	73

[*]Locus tags to address proteins in genome of the strain H9 (GCA_000525715.1)

**Assigned to GH by manual BLASTp analysis

Supplemental Table S4. continued.

	H10		
Locus tag[*]	Annotated as	Signal peptide	GH
GCA000189515.1_01413	6-phospho-beta-glucosidase	N	1-1
GCA000189515.1_01585-01586	6-phospho-beta-glucosidase	N	1-2
GCA000189515.1_01426	6-phospho-beta-glucosidase	N	1-3
GCA000189515.1_01409	6-phospho-beta-glucosidase	N	1-5
GCA000189515.1_00711	beta-galactosidase	N	2
GCA000189515.1_01980	6-phospho-alpha-glucosidase	N	4
GCA000189515.1_00541	alpha, alpha-phosphotrehalase	N	13-4
GCA000189515.1_02207	alpha-glucosidase	N	13-5
GCA000189515.1_02206	alpha-amylase	N	13-7
GCA000189515.1_00861	lysin	Y (1-26)	25
GCA000189515.1_02247	lysin	Y (1-36)	25
GCA000189515.1_01045	lysin	N	25
GCA000189515.1_01704	lysozyme	N	25
GCA000189515.1_00169	alpha-glucosidase	N	31-1
GCA000189515.1_02090	alpha-glucosidase	N	31-2
GCA000189515.1_00427	sucrose-6-phosphate hydrolase	N	32-1
GCA000189515.1_01880	6-phospho-alpha-glucosidase	N	32-2
GCA000189515.1_01876	alpha-galactosidase	N	36-1
GCA000189515.1_00718[**]	beta-galactosidase	N	42
GCA000189515.1_02205	maltose phosphorylase	N	65-1
GCA000189515.1_00203	N-acetylmuramidase	Y (1-23)	73
GCA000189515.1_01805	N-acetylmuramidase	N	73

[*]Locus tags to address proteins in genome of the strain H10 (GCA000189515.1)

**Assigned to GH by manual BLASTp analysis

Supplemental Table S4. continued.

	KLDS1.8701		
Locus tag*	Annotated as	Signal peptide	GH
GCA000961015.1_01591	6-phospho-beta-glucosidase	N	1-1
GCA000961015.1_01798-01800	6-phospho-beta-glucosidase	N	1-2
GCA000961015.1_01601-01603	6-phospho-beta-glucosidase	N	1-3
GCA000961015.1_01586	6-phospho-beta-glucosidase	N	1-5
GCA000961015.1_00926	beta-galactosidase	N	2
GCA000961015.1_00654	beta-N-acetylhexosaminidase	N	3
GCA000961015.1_00334	beta-glucanase	N	8
GCA000961015.1_01062	lysin	Y (1-26)	25
GCA000961015.1_00113	lysin	Y (1-36)	25
GCA000961015.1_01270	lysin	N	25
GCA000961015.1_01933	lysozyme	N	25
GCA000961015.1_00373	alpha-glucosidase	N	31-1
GCA000961015.1_00933	beta-galactosidase	N	42
GCA000961015.1_00406	N-acetylmuramidase	Y (1-23)	73
GCA000961015.1_02022	N-acetylmuramidase	N	73

*Locus tags to address proteins in genome of the strain KLDS1.8701 (GCA_000961015.1)

Supplemental Table S4. continued.

	MB2-1		
Locus tag*	Annotated as	Signal peptide	GH
GCA_001006025.1_01022-01023**	6-phospho-beta-glucosidase	N	1-1
GCA001006025.1_00822-00824	phospho-beta-galactosidase II	N	1-2
GCA001006025.1_01029-01030	6-phospho-beta-glucosidase	N	1-5
GCA001006025.1_01868	beta-galactosidase	N	2
GCA001006025.1_00130	beta-glucanase	N	8
GCA001006025.1_01085	SLT domain protein	N	23
GCA001006025.1_01466	lysin	N	25
GCA001006025.1_01643	hypothetical protein	N	25
GCA001006025.1_01738	lysin	Y (1-26)	25
GCA001006025.1_02345	lysin	Y (1-36)	25
GCA001006025.1_01396	lysin	N	25
GCA001006025.1_00709	lysozyme	N	25
GCA001006025.1_00166	alpha-glucosidase	N	31-1
GCA001006025.1_00521	alpha-galactosidase 1-like protein	N	32
GCA001006025.1_01860**	beta-galactosidase	N	42
GCA001006025.1_00199	N-acetylmuramidase	Y (1-23)	73
GCA001006025.1_00609	N-acetylmuramidase	N	73

*Locus tags to address proteins in genome of the strain MB2-1 (GCA_001006025.1)

**Assigned to GH by manual BLASTp analysis

Supplemental Table S4. continued.

	R0052		
Locus tag*	Annotated as	Signal peptide	GH
GCA000165775.3_01427-01428	6-phospho-beta-glucosidase	N	1-1
GCA000165775.3_01598	6-phospho-beta-glucosidase	N	1-2
GCA000165775.3_01442	6-phospho-beta-glucosidase	N	1-3
GCA000165775.3_00630	6-phospho-beta-glucosidase	N	1-4
GCA000165775.3_00758	beta-galactosidase	N	2
GCA000165775.3_00455	beta-N-acetylhexosaminidase	Y (1-31)	3
GCA000165775.3_01935	6-phospho-alpha-glucosidase	N	4
GCA000165775.3_00126	beta-glucanase	N	8
GCA000165775.3_01227	alpha, alpha-phosphotrehalase	N	13-3
GCA000165775.3_02229	alpha-glucosidase	N	13-5
GCA000165775.3_01834	sucrose phosphorylase	N	13-6
GCA000165775.3_02228	alpha-amylase	N	13-7
GCA000165775.3_01639	amylopullulanase	N	13-8
GCA000165775.3_01644	glycogen branching enzyme	N	13-9
GCA000165775.3_01077	lysin	Y (1-39)	25
GCA000165775.3_00881	lysin	Y (1-26)	25
GCA000165775.3_02291	lysin	Y (1-36)	25
GCA000165775.3_01722	lysozyme	N	25
GCA000165775.3_00161	alpha-glucosidase	N	31-1
GCA000165775.3_02089	alpha-glucosidase	N	31-2
GCA000165775.3_00436	sucrose-6-phosphate hydrolase	N	32-1
GCA000165775.3_00764	beta-galactosidase	N	42
GCA000165775.3_02227	maltose phosphorylase	N	65-1
GCA000165775.3_00195	N-acetylmuramidase	Y (1-23)	73
GCA000165775.3_01812	N-acetylmuramidase	Y (1-38)	73

*Locus tags to address proteins in genome of the strain R0052 (GCA_000165775.3)

Supplemental Table S4. continued.

	H-8		
Locus tag*	Annotated as	Signal peptide	GH
LHEH8_13940	6-phospho-beta-glucosidase	N	1-1
LHEH8_06370	6-phospho-beta-glucosidase	N	1-2
LHEH8_13100	6-phospho-beta-glucosidase	N	1-3
LHEH8_07500	6-phospho-beta-glucosidase	N	1-4
LHEH8_13900	6-phospho-beta-glucosidase	N	1-5
LHEH8_01180	beta-galactosidase	N	2
LHEH8_14580	6-phospho-alpha-glucosidase	N	4
LHEH8_15840	beta-glucanase	N	8
LHEH8_10170	oligo-1,6-glucosidase	N	13-1
LHEH8_16900	oligo-1,6-glucosidase	N	13-2
LHEH8_16980	alpha-glucosidase	N	13-4
LHEH8_11710	sucrose phosphorylase	N	13-6
LHEH8_16990	alpha-amylase	N	13-7
LHEH8_12710	hypothetical protein	N	13-9
LHEH8_08750	lysin	Y (1-26)	25
LHEH8_16490	lysin	Y (1-36)	25
LHEH8_18820	lysozyme	N	25
LHEH8_05750	alpha-glucosidase	N	31-1
LHEH8_04270	alpha-glucosidase	N	31-2
LHEH8_16920	glycosyl hydrolase family 31	N	31-3
LHEH8_18460	alpha-glucosidase	N	31-4
LHEH8_17780	sucrose-6-phosphate hydrolase	N	32-1
LHEH8_18690	alpha-galactosidase	N	36-2
LHEH8_17000	maltose phosphorylase	N	65-1
LHEH8_09090	N-acetylmuramidase	N	73
LHEH8_12290	N-acetylmuramidase	Y (1-23)	73

*Locus tags to address proteins in genome of the strain H-8 (BLYO01000001)

Supplemental Table S4. continued.

	JCM 30912		
Locus tag*	Annotated as	Signal peptide	GH
ERR387534_01726	6-phospho-beta-glucosidase	N	1-1
ERR387534_01375	6-phospho-beta-glucosidase	N	1-2
ERR387534_00736	6-phospho-beta-glucosidase	N	1-3
ERR387534_01540	6-phospho-beta-glucosidase	N	1-4
ERR387534_00524	6-phospho-beta-glucosidase	N	1-5
ERR387534_00608	beta-galactosidase	N	2
ERR387534_01413	6-phospho-alpha-glucosidase	N	4
ERR387534_01626	beta-glucanase	N	8
ERR387534_01309	oligo-1,6-glucosidase	N	13-1
ERR387534_01525	oligo-1,6-glucosidase	N	13-2
ERR387534_00580	alpha-glucosidase	N	13-5
ERR387534_01042	sucrose phosphorylase	N	13-6
ERR387534_00579	alpha-amylase	N	13-7
ERR387534_00969	glycogen branching enzyme	N	13-9
ERR387534_01704	lysin	Y (1-26)	25
ERR387534_00882	lysin	Y (1-36)	25
ERR387534_00830	lysozyme	N	25
ERR387534_01681	alpha-glucosidase	N	31-1
ERR387534_01280	alpha-glucosidase	N	31-2
ERR387534_00586	glycosyl hydrolase family 31	N	31-3
ERR387534_01486	alpha-glucosidase	N	31-4
ERR387534_01231	sucrose-6-phosphate hydrolase	N	32-1
ERR387534_01593	alpha-galactosidase	N	36-2
ERR387534_01349	beta-galactosidase	N	42
ERR387534_00578	maltose phosphorylase	N	65-1
ERR387534_00768	N-acetylmuramidase	N	73
ERR387534_01092	N-acetylmuramidase	Y (1-23)	73

*Locus tags to address proteins in genome of
the strain JCM 30912 (LMG22464; accession no. ERR387534)

Supplemental Table S4. continued.

	LMG 22465		
Locus tag*	Annotated as	Signal peptide	GH
LMG22465_01059	6-phospho-beta-glucosidase	N	1-1
LMG22465_01269	6-phospho-beta-glucosidase	N	1-2
LMG22465_00986	6-phospho-beta-glucosidase	N	1-3
LMG22465_01697	6-phospho-beta-glucosidase	N	1-4
LMG22465_00131	6-phospho-beta-glucosidase	N	1-5
LMG22465_01279	beta-galactosidase	N	2
LMG22465_00237	6-phospho-alpha-glucosidase	N	4
LMG22465_00690	beta-glucanase	N	8
LMG22465_00460	oligo-1,6-glucosidase	N	13-1
LMG22465_00122	oligo-1,6-glucosidase	N	13-2
LMG22465_00050	alpha-glucosidase	N	13-5
LMG22465_01368	sucrose phosphorylase	N	13-6
LMG22465_00051	alpha-amylase	N	13-7
LMG22465_01313	glycogen branching enzyme	N	13-9
LMG22465_01785	lysin	N	25
LMG22465_01412	lysin	Y (1-36)	25
LMG22465_00274	lysozyme	N	25
LMG22465_01410	alpha-glucosidase	N	31-1
LMG22465_01505	alpha-glucosidase	N	31-2
LMG22465_00044	glycosyl hydrolase family 31	N	31-3
LMG22465_00650	alpha-glucosidase	N	31-4
LMG22465_00614	sucrose-6-phosphate hydrolase	N	32-1
LMG22465_00461	alpha-galactosidase	N	36-2
LMG22465_00955	beta-galactosidase	N	42
LMG22465_00052	maltose phosphorylase	N	65-1
LMG22465_00775	N-acetylmuramidase	N	73
LMG22465_01730	N-acetylmuramidase	Y (1-23)	73

*Locus tags to address proteins in genome of the strain LMG 22465 (BLYR01000001)

Supplemental Table S4. continued.

	W-6		
Locus tag[*]	Annotated as	Signal peptide	GH
LHEW6_15540	6-phospho-beta-glucosidase	N	1-1
LHEW6_14650	6-phospho-beta-glucosidase	N	1-2
LHEW6_04130	6-phospho-beta-glucosidase	N	1-3
LHEW6_06390	6-phospho-beta-glucosidase	N	1-4
LHEW6_15500	6-phospho-beta-glucosidase	N	1-5
LHEW6_13340	beta-galactosidase	N	2
LHEW6_14980	6-phospho-alpha-glucosidase	N	4
LHEW6_08490	beta-glucanase	N	8
LHEW6_00720	oligo-1,6-glucosidase	N	13-1
LHEW6_01920	oligo-1,6-glucosidase	N	13-2
LHEW6_17030	alpha-glucosidase	N	13-5
LHEW6_15970	sucrose phosphorylase	N	13-6
LHEW6_17040	alpha-amylase	N	13-7
LHEW6_14710	hypothetical protein	N	13-9
LHEW6_10040	lysin	Y (1-26)	25
LHEW6_00860	lysozyme	N	25
LHEW6_03130	alpha-glucosidase	N	31-2
LHEW6_01940	glycosyl hydrolase family 31	N	31-3
LHEW6_07170	alpha-glucosidase	N	31-4
LHEW6_08410	sucrose-6-phosphate hydrolase	N	32-1
LHEW6_00730	alpha-galactosidase	N	36-2
JHEW6_06755[**]	maltose phosphorylase	N	65-1
LHEW6_13270	N-acetylmuramidase	N	73
LHEW6_17900	N-acetylmuramidase	Y (1-23)	73

[*]Locus tags to address proteins in genome of the strain W-6 (BLYP01000001)

**Assigned to GH by manual BLASTp analysis

Supplemental Table S4. continued.

	Y-10		
Locus tag*	Annotated as	Signal peptide	GH
LHEY10_08080	6-phospho-beta-glucosidase	N	1-1
LHEY10_10390	6-phospho-beta-glucosidase	N	1-2
LHEY10_03180	6-phospho-beta-glucosidase	N	1-3
LHEY10_07700	6-phospho-beta-glucosidase	N	1-4
LHEY10_08040	beta-glucosidase	N	1-5
LHEY10_03890	beta-galactosidase large subunit	N	2
LHEY10_11800	maltose-6'-phosphate glucosidase	N	4
LHEY10_11650	beta-glucanase	N	8
LHEY10_12280	oligo-1,6-glucosidase	N	13-1
LHEY10_16510	oligo-1,6-glucosidase	N	13-2
LHEY10_16590	oligo-1,6-glucosidase	N	13-5
LHEY10_15170	sucrose phosphorylase	N	13-6
LHEY10_16600	alpha-glycosidase	N	13-7
LHEY10_17950	hypothetical protein	N	13-9
LHEY10_10790	lysin	Y (1-26)	25
LHEY10_01330	lysin	Y (1-36)	25
LHEY10_12420	lysozyme	N	25
LHEY10_11750	alpha-glucosidase	N	31-1
LHEY10_01540	alpha-glucosidase	N	31-2
LHEY10_16530	glycosyl hydrolase	N	31-3
LHEY10_17450	alpha-glucosidase	N	31-4
LHEY10_09110	invertase	N	32-1
LHEY10_12290	alpha-galactosidase	N	36-2
LHEY10_16610	maltose phosphorylase	N	65-1
LHEY10_07250	N-acetylmuramidase	Y (1-23)	73
LHEY10_15840	N-acetylmuramidase	N	73

*Locus tags to address proteins in genome of the strain Y-10 (BLYQ01000001)

Supplemental materials

Fig. S1. Genome-wide BLAST comparison of all *L. helveticus* strains against reference strain CNRZ32. Blue regions indicate specific genomic regions in CNRZ32 and red regions are specific regions in milk strains. Gray regions are pseudo-specific regions.

Fig. S2. Hierarchical clustering of *L. helveticus* strains based on the numbers of GH family proteins. Numbers of estimated proteins in each GH family were used to prepare a dendrogram using the hclust function with the Ward.D2 algorithm in the R package (version 3.6.2). Strain names of the whisky strains are indicated in blue and those of the milk strains are in orange.

Fig. S3. Phylogenetic relationships of GH4 enzymes in *L. helveticus* strains. Locus tags and strain names are shown. Strain names of the whisky strains are indicated in blue and those of the milk strains are in orange. GH2 enzyme of *L. helveticus* JCM 1120^T (LHEJCM1120_13990) was used as an outgroup. Bootstrap percentages above 90% are indicated at branching points.

Fig. S4. Phylogenetic relationships of GH65 enzymes in *L. helveticus* strains. Locus tags and strain names are shown. Strain names of the whisky strains are indicated in blue and those of the milk strains are in orange. Reference GH65 enzyme in *L. acidophilus* NCFM is shown in red. GH2 enzyme of *L. helveticus* JCM 1120^T (LHEJCM1120_13990) was used as an outgroup. Bootstrap percentages above 90% are indicated at branching points.

Fig. S5. Phylogenetic relationships of GH36 enzymes in *L. helveticus* strains. Locus tags and strain names are shown. Strain names of the whisky strains are indicated in blue and those of the milk strains are in orange. Reference GH36 enzyme in *L. acidophilus* NCFM is shown in red. GH2 enzyme of *L. helveticus* JCM 1120^T (LHEJCM1120_13990) was used as an outgroup. Bootstrap percentages above 90% are indicated at branching points.

Fig. S6. Phylogenetic relationships of GH31 enzymes in *L. helveticus* strains. Locus tags and strain names are shown. Strain names of the whisky strains are indicated in blue and those of the milk strains are in orange. GH2 enzyme of *L. helveticus* JCM 1120^T (LHEJCM1120_13990) was used as an outgroup. Bootstrap percentages above 90% are indicated at branching points.

Supplemental Fig. S1

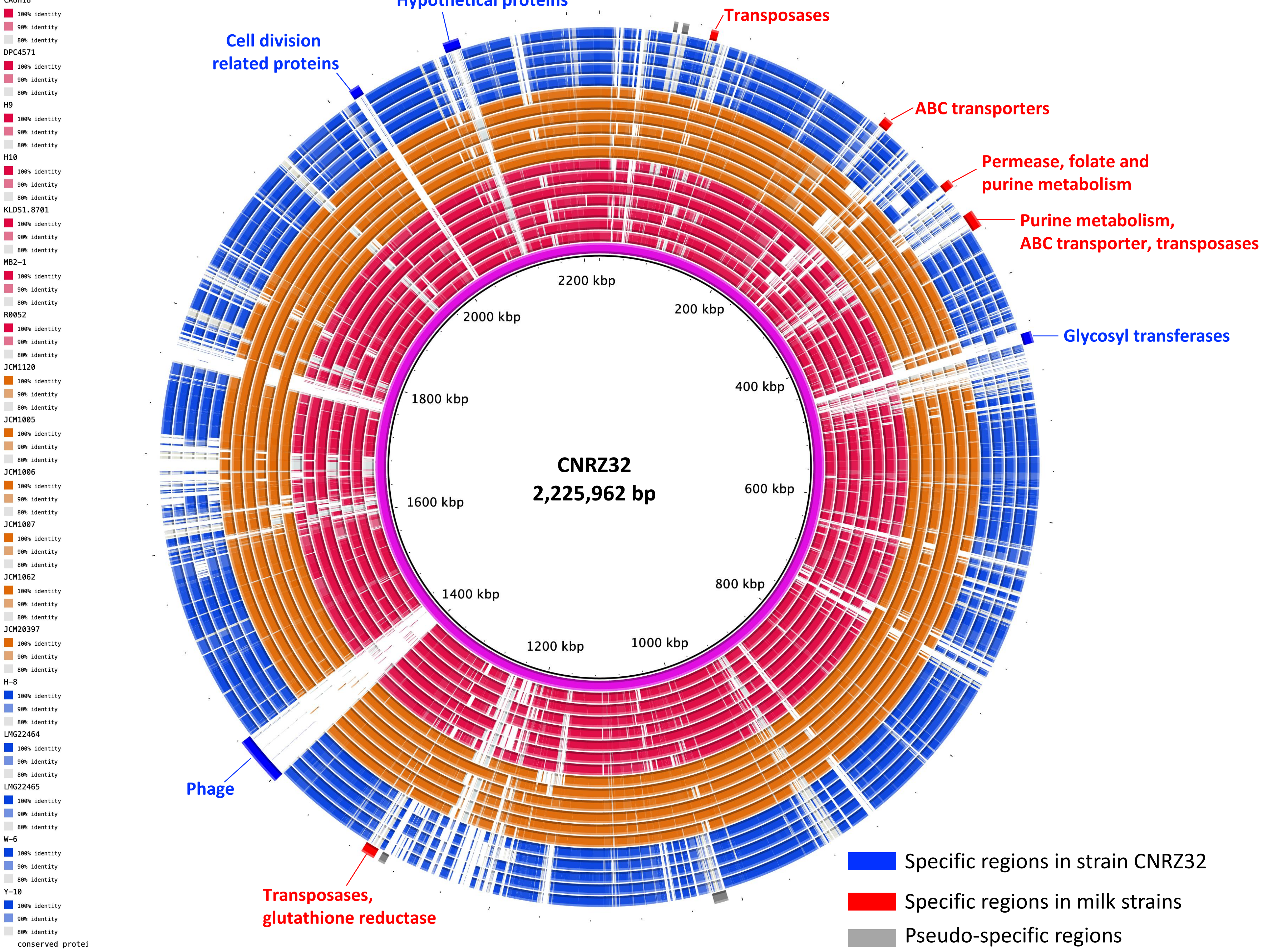

Supplemental Fig. S2

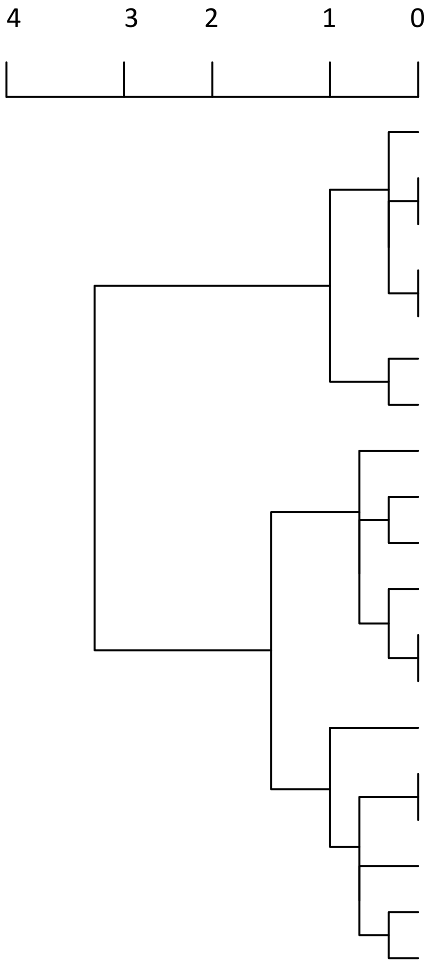

	GH1	GH2	GH3	GH4	GH8	GH13	GH23	GH25	GH31	GH32	GH36	GH42	GH65	GH68	GH73
W-6	5	1	0	1	1	6	0	2	3	1	1	0	1	0	2
JCM 30912	5	1	0	1	1	6	0	3	4	1	1	1	1	0	2
LMG 22465	5	1	0	1	1	6	0	3	4	1	1	1	1	0	2
H-8	5	1	0	1	1	6	0	3	4	1	1	0	1	0	2
Y-10	5	1	0	1	1	6	0	3	4	1	1	0	1	0	2
CAUH18	4	1	0	1	0	6	0	4	2	2	1	1	1	0	2
R0052	4	1	1	1	1	6	0	4	2	1	0	1	1	0	2
MB2-1	3	1	0	0	1	0	1	6	1	1	0	1	0	0	2
KLDS 1.8701	4	1	1	0	1	0	0	4	1	0	0	1	0	0	2
JCM 1005	4	1	1	0	1	0	0	4	1	0	0	1	0	1	2
H9	4	1	0	0	1	1	0	4	1	1	0	1	0	0	2
JCM 1006	3	1	0	0	1	0	0	4	1	1	1	1	0	0	2
JCM 20397	3	1	0	0	1	0	0	4	1	1	1	1	0	0	2
H10	4	1	0	1	0	3	0	4	2	2	1	1	1	0	2
JCM 1007	4	1	1	0	1	2	0	4	1	0	0	1	2	0	2
JCM 1062	4	1	1	0	1	2	0	4	1	0	0	1	2	0	2
DPC 4571	4	1	0	0	0	2	0	4	1	0	1	1	1	0	2
CNRZ32	4	1	1	0	1	3	0	5	1	0	0	1	1	0	2
JCM 1120^T	4	1	0	1	1	3	0	4	1	0	0	1	1	0	2

Supplemental Fig. S3

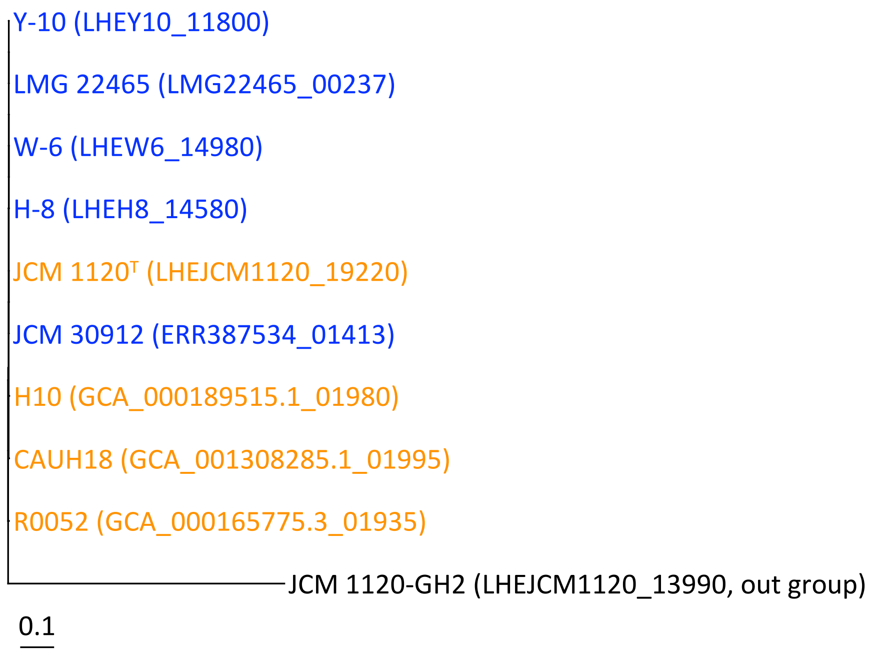

Supplemental Fig. S4

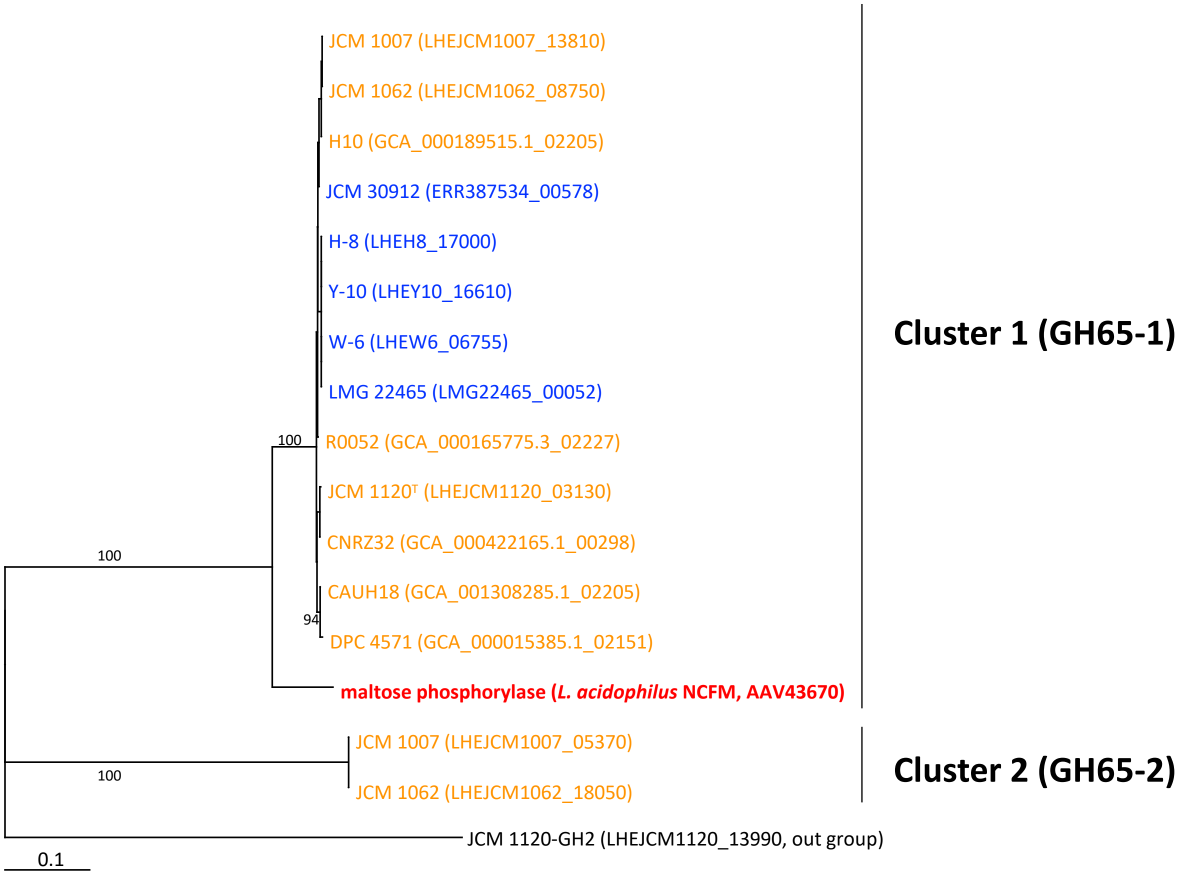

Supplemental Fig. S5

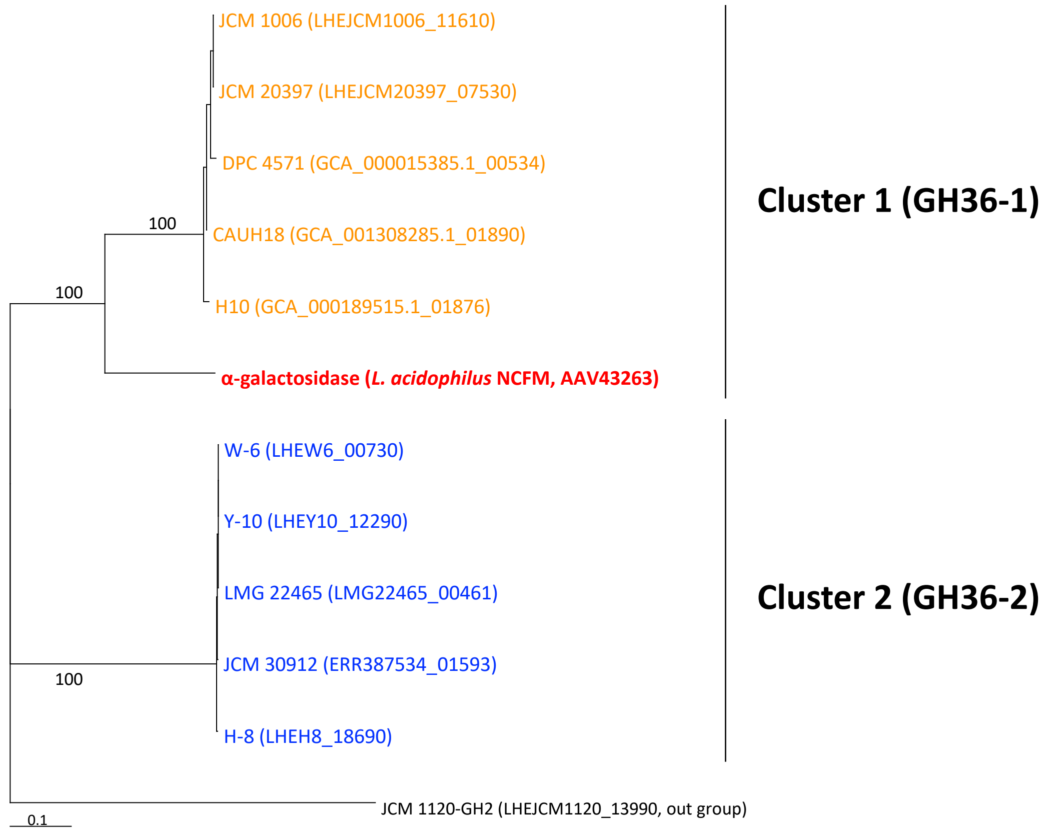

Supplemental Fig. S6

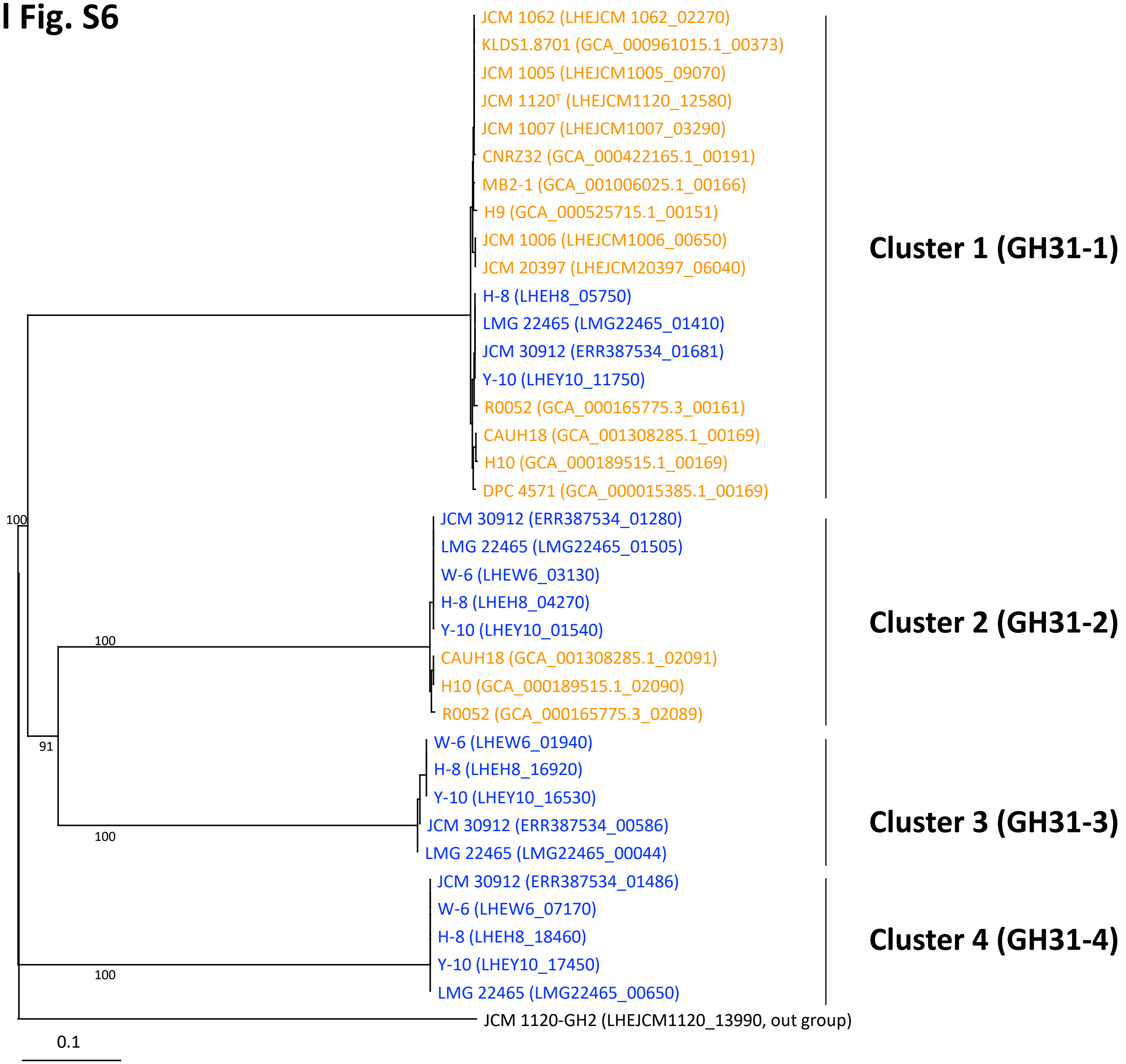

Supplement: Supplementary material 1 [file mgen-7-0560-s001.pdf]
